# A Comprehensive Review of Advances in Magnesium-Based Cementitious Materials: Hydration, Properties, and Applications in Soil Stabilization

**DOI:** 10.3390/ma18163806

**Published:** 2025-08-13

**Authors:** Qi Xu, Dongliang Chen, Jian Xiong, Xin He, Shengde Dong, Luxiang Ma, Chunxi Hai, Yuan Zhou, Yanxia Sun

**Affiliations:** 1 College of Materials and Chemistry & Chemical Engineering, Chengdu University of Technology, Chengdu 610059, China; 2 Department of Biological and Chemical Engineering, Agricultural School, Panzhihua University, Panzhihua 617000, China

**Keywords:** magnesium-based cementitious materials, hydration characteristics, properties, applications, research progress

## Abstract

This review provides a comprehensive overview of the advancements in magnesium-based cementitious materials (MBCMs), including magnesium oxychloride cementitious material (MOC), magnesium oxysulfate cementitious material (MOS), and magnesium phosphate cementitious material (MPC). The hydration processes and products, performance characteristics, and applications in soil stabilization are systematically discussed. Key findings reveal that MOC exhibits rapid strength development and excellent thermal stability, while MOS demonstrates improved water resistance and mechanical properties. MPC is highlighted for its effectiveness in the immobilization of heavy metals. The environmental impact of MBCMs is also evaluated, highlighting their potential for sustainable development in civil engineering applications. The primary issues and challenges for MBCMs in soil curing include the insufficient stability of hydration products and inadequate understanding of curing mechanisms, leading to variable material properties and difficulties in precisely controlling the curing effects in practical engineering. Additionally, the complex composition of MBCMs and the highly variable characteristics of natural soils result in significant differences in curing effectiveness under different conditions, restricting their application scope and posing risks to project costs and quality stability.

## 1. Introduction

Magnesium-based cement is a novel type of cementitious material, primarily composed of active magnesium oxide (MgO). It can be categorized into three main types: magnesium oxychloride cementitious material (MOC) based on the MgO-MgCl_2_-H_2_O ternary system, magnesium oxysulfate cementitious material (MOS) based on the MgO-MgSO_4_-H_2_O system, and magnesium phosphate cementitious material (MPC) based on the MgO-PO_4_^3−^-H_2_O system.

**Figure 1:**
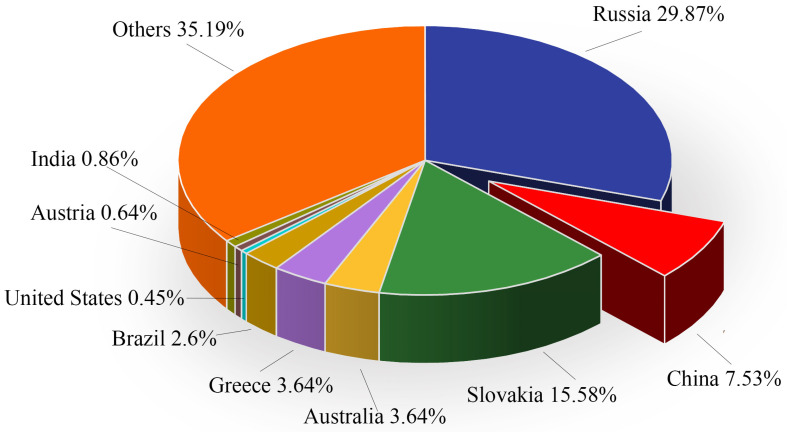
Proportion of global magnesite reserves by country in 2023 (Data Source: USGS, Tianfeng Securities Research Institute).

Light-burned magnesia, a critical raw material for magnesium-based cementitious materials, is produced by calcining magnesite (primarily composed of MgCO_3_) at temperatures between 750 °C and 900 °C [1]. Magnesite is an abundant mineral resource in China, ranking first globally in terms of reserves, production, and export volume [2]. According to data from the United States Geological Survey (USGS), global magnesite reserves in 2023 were estimated at 7.7 billion tons, with China holding 580 million tons, accounting for 7.53% of the global total and ranking third worldwide. The distribution of magnesite reserves among countries in 2023 is illustrated in Figure 1. Additionally, the process of extracting lithium carbonate from salt lake brine often produces a significant amount of milky white block waste residue, primarily composed of Mg(OH)_2_, MgO, and Mg_2_B_3_O_6_ [3]. This abundant supply of raw materials provides a solid foundation for large-scale magnesium cement production, offering a significant advantage in resource acquisition and ensuring its potential for sustainable development.

Magnesium-based cement exhibits superior performance in environmental protection and energy conservation compared to traditional Portland cement. The production of Portland cement involves a “two grinding and one burning” process, with the calcination temperature of clinker reaching 1400 °C to 1500 °C. In contrast, the calcination temperature for light-burned MgO in magnesium-based cement production is only 750 °C to 900 °C, and the coal consumption is approximately 71 kg/t. This not only reduces energy consumption but also significantly decreases CO_2_ emissions during the production process. Compared to Portland cement, magnesium cement can reduce CO_2_ emissions by 30% to 50% [4,5]. In the context of global efforts to promote green buildings and low-carbon development, this environmentally friendly and energy-efficient characteristic aligns well with the construction policy of “applicability, economy, greenness, and aesthetics” [6]. As environmental protection standards become increasingly stringent, the construction industry is placing greater emphasis on the environmental friendliness of building materials. Magnesium-based cementitious materials, with their environmental and energy-saving advantages, should gradually replace some general-purpose Portland cement and become an important force in the green transformation of the building materials sector. This shift is crucial for promoting the sustainable development of the entire construction industry.

**Figure 2:**
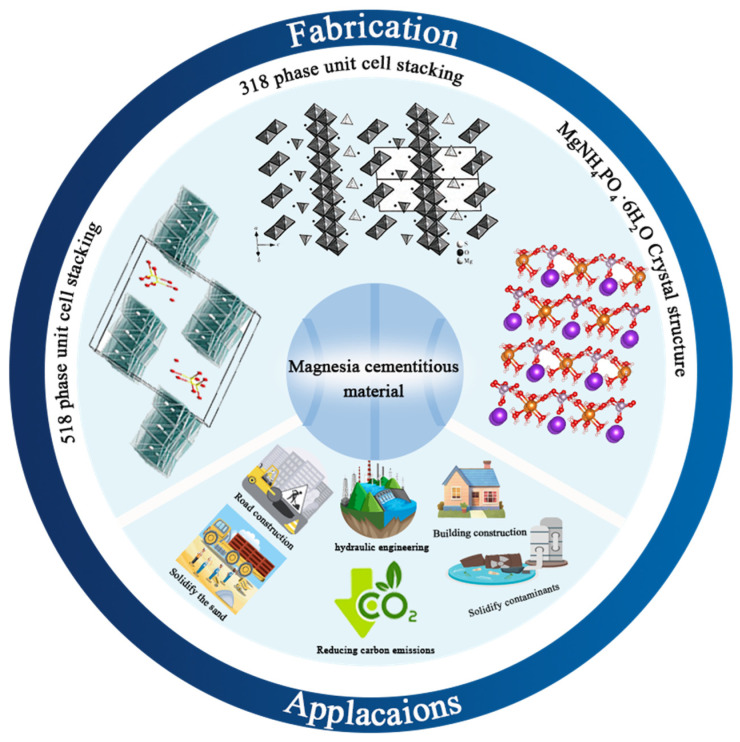
Structure and applications of magnesium-based cementitious materials (Overall composition from [11]; crystal structure diagrams from [12,13,14]).

Magnesium-based cementitious materials have found extensive applications in various critical fields due to their unique properties, as illustrated in Figure 2. In contaminated soil remediation, they can chemically react with or physically adsorb harmful substances in polluted soil, effectively reducing pollution levels and improving soil quality, thereby enabling the reuse of contaminated land or mitigating environmental risks [7]. In soil stabilization, they enhance the strength and stability of soil, preventing deformation and landslides in projects such as road subgrades and slope protection, ensuring the safety and durability of these structures [8]. In the building materials sector, they are used to manufacture various building components and decorative materials, leveraging their mechanical properties while meeting environmental protection requirements, thus providing more green options for the building materials market [9]. In nuclear waste immobilization, they effectively encapsulate and isolate nuclear waste, preventing radiation leakage and protecting the environment and human health, thanks to their corrosion resistance and stability [10]. These diverse applications highlight the versatility and importance of magnesium-based cementitious materials in modern engineering technology and environmental protection.

Given the significant advantages of magnesium-based cementitious materials in raw material reserves, environmental protection, energy conservation, and application fields, as well as the growing importance of soil stabilization in numerous engineering and environmental sectors, this paper focuses on the application background of soil stabilization. It provides a detailed review of research progress on the three main types of magnesium-based cementitious materials for soil stabilization both domestically and internationally. Through systematic analysis of existing research results, the aim is to comprehensively understand the current status, technical characteristics, challenges, and development trends of magnesium-based cementitious materials in soil stabilization. This will provide theoretical foundations and practical references for optimizing their application in soil stabilization, promoting technological innovation and sustainable development in related fields. Additionally, it aims to build a bridge for information exchange and knowledge inheritance for future research and engineering practice, driving the entire industry towards higher standards in soil stabilization technology and the application of magnesium-based cementitious materials.

## 2. Methodology

In this literature review, some inclusion criteria for literature review research were defined. For example, the language chosen was English and Chinese for the following reasons: first, English serves as the universal language in global scientific research, ensuring access to mainstream academic achievements and international perspectives. Second, China’s unique geological endowment—characterized by extensive loess regions and abundant magnesium resources (including magnesite ore and salt lake magnesium deposits)—has fostered prolific research in two key domains, namely soil solidification technologies adapted to loess engineering challenges and magnesium-based cementitious materials leveraging local mineral resources. Focusing on the Chinese-language literature is essential to capture these region-specific research dynamics, which are critical for a comprehensive analysis of soil improvement and sustainable cementitious material development.

The databases MDPI, ScienceDirect, Springer, Web of Science, and CNKI (China National Knowledge Infrastructure) were chosen, as they host abundant scientific journals and periodicals. Relevant documents were then retrieved, including review articles, research papers, and academic dissertations. The data selection and collection processes were carried out in May 2025, and in the first step of the process, the keywords used in the abovementioned databases were identified, such as “magnesium oxychloride cement” or “magnesium oxysulfate cement” or “magnesium phosphate cement” or “cementitious materials” or “hydration characteristics” or “soil stabilization” or “loess curing” or “stabilization technology”. In the second stage, the filters available in the databases were applied. For example, the periods of the research years, the types of papers, the types of publication, and the subject areas.

The selected papers contain information on magnesium-based cementitious materials, focusing on several materials like magnesium oxychloride cement, magnesium oxysulfate cement, and magnesium phosphate cement. This study will focus on the hydration mechanism and application performance of magnesium-based cement in soil solidification. Therefore, general contents are considered, such as hydration products, performance, and application in soil solidification. Papers that include other approaches were excluded from this study.

## 3. Soil Stabilization Materials

Soil, as a critical natural resource on Earth, is intrinsically linked to agriculture and human development and plays an indispensable role in maintaining ecosystem balance and supporting human survival. However, the exploitation and utilization of soil resources have led to numerous severe issues due to excessive development, frequent engineering activities, and the rapid advancement of industrialization and urbanization. These issues include soil erosion, soil subsidence, soil pollution, and desertification. Such problems not only impose significant pressure on the ecological environment but also pose substantial challenges to the sustainable development of human society.

**Figure 3:**
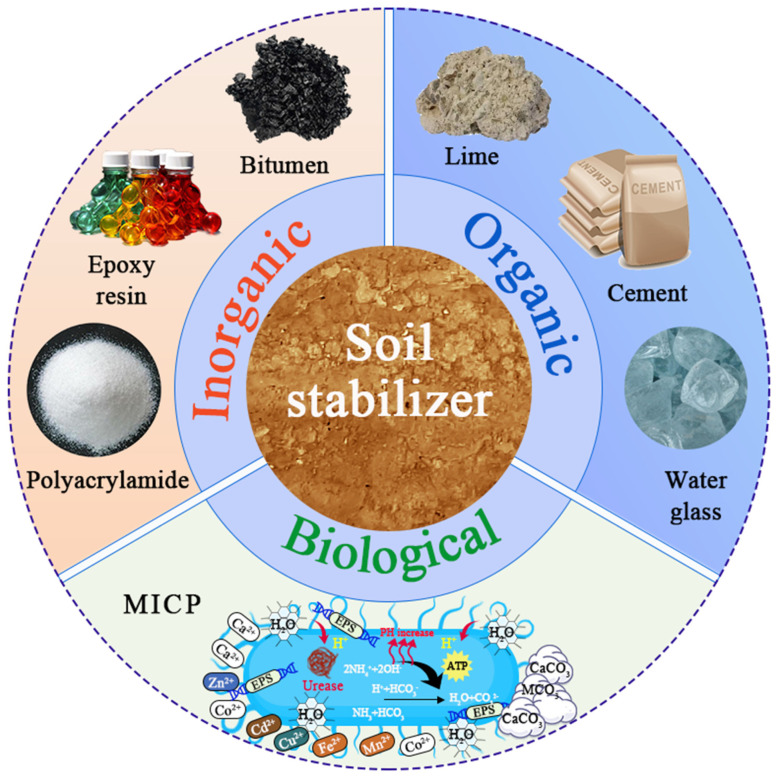
Common types of soil stabilizers (MICP mechanism diagram is derived from [22]).

Soil stabilization technology, as a highly promising solution, has demonstrated significant advantages and value across various fields, including geology [15], water resource management [16], land development [17], specialized engineering construction [18,19], and soil pollution remediation [20,21]. It offers innovative approaches to addressing the aforementioned challenges. Soil stabilization technology involves the process of binding soil particles using physical, chemical, and biological methods to enhance soil strength, reduce deformation, and decrease permeability. The choice and effectiveness of stabilizers are critical in this process. Commonly used soil stabilizers can be categorized into three main types, which are inorganic stabilizers, organic stabilizers, and biological stabilizers, as illustrated in Figure 3.

Table 1 summarizes studies on the solidification mechanism, namely the solidification effect of various soil stabilization materials. Among the three types of stabilizers discussed, each has distinct advantages in enhancing the bearing capacity and anti-permeability of different soil types. Organic stabilizers are generally expensive, biological stabilizers have stringent environmental requirements, while inorganic stabilizers, especially cement-based ones, offer good economic benefits [23]. However, traditional cement-based stabilizers also pose environmental challenges, including CO_2_ emissions, the depletion of natural resources, and high energy consumption during production. For instance, in the production of Portland cement (containing 95% clinker), limestone calcination releases 0.513 kg of CO_2_ per kilogram of cement, and producing each kilogram of clinker requires 1.67 MJ of energy. Moreover, the cement industry consumes 12–15% of total industrial energy [24,25]. Therefore, exploring more sustainable and environmentally friendly soil stabilization solutions has become a critical research focus.

## 4. Magnesium Oxychloride Cementitious Material

### 4.1. Hydration Products of MOC

In 1867, Sorel first discovered that mixing active MgO with a certain concentration of MgCl_2_ solution could produce an air-hardening magnesium-based cementitious material—magnesium oxychloride cement [41]. Under normal temperature and pressure, the primary hydration products in the MOC system are 3Mg(OH)_2_·MgCl_2_·8H_2_O (3·1·8 phase), 5Mg(OH)_2_·MgCl_2_·8H_2_O (5·1·8 phase), and Mg(OH)_2_. The formation process is illustrated in Equations (1) and (2).

(1) $$5MgO+{Mg}^{2+}+2{Cl}^{-}+13H_{2}O\to 5{Mg(OH)}_{2}\cdot {MgCl}_{2}\cdot 8H_{2}O$$

(2) $$3MgO+{Mg}^{2+}+2{Cl}^{-}+11H_{2}O\to 3{Mg(OH)}_{2}\cdot {MgCl}_{2}\cdot 8H_{2}O$$

**Figure 4:**
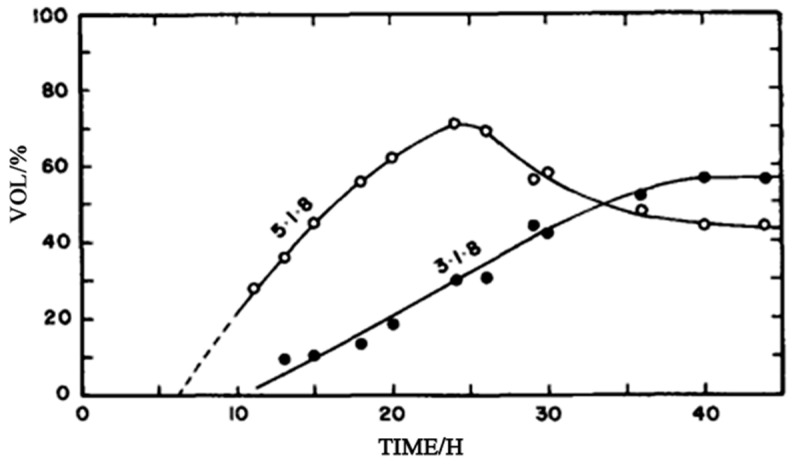
Relative amounts of 5·1·8 and 3·1·8 phases observed as a function of time in sample 8 (sample 8: 1.0–1.2% MgO, 22.2–34.5% MgCl_2_, and the remaining H_2_O) [42].

In 1980, Urwongse [42] reported that compared with other phases, the 5·1·8 phase, as a metastable intermediate product, rapidly forms and reaches its peak within the initial reaction stage (approximately 25 h). The 3·1·8 phase gradually becomes the dominant phase by slowly consuming the 5·1·8 phase, finally achieving equilibrium in a closed container after about 10 days, as shown in Figure 4. In early studies, the stable region of the 5·1·8 phase was overestimated due to insufficient waiting for equilibrium. Meanwhile, in practical applications, the delayed effect of the transformation from the 5·1·8 phase to the 3·1·8 phase should also be considered. Additionally, the 3·1·8 phase is prone to react with atmospheric CO_2_ to form chlorocarbonate, which has a significant impact on the surface stability of materials.

**Figure 5:**
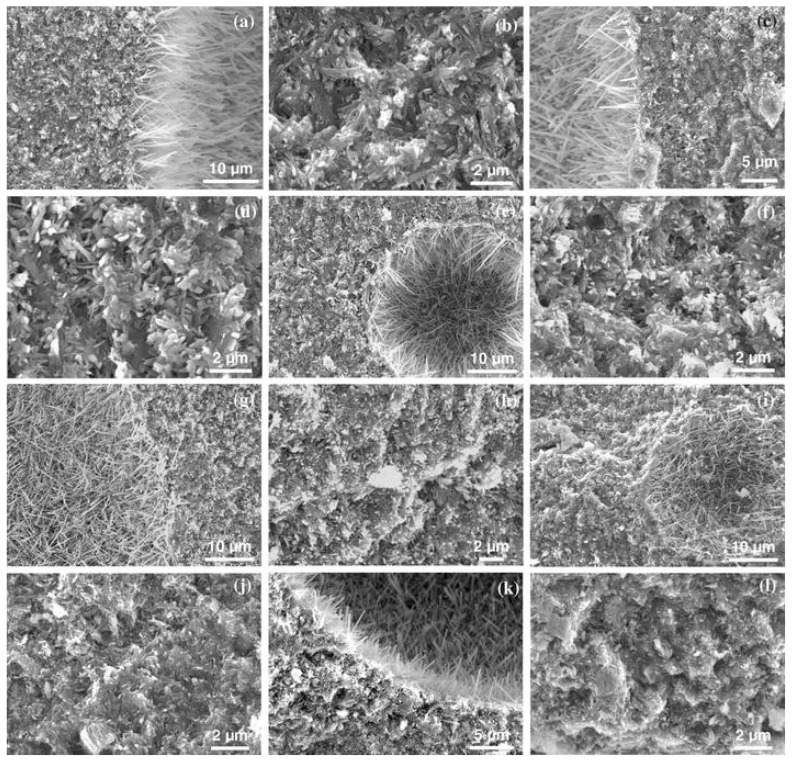
Microscopic morphology of hydration products in magnesium oxychloride cement after 7 days of curing. (**a**,**b**) Composition A (MgO 38.0 wt%, MgCl_2_·6H_2_O 38.3 wt%, deionized water 23.7 wt%, MgO:MgCl_2_ molar ratio 5, H_2_O:MgCl_2_ molar ratio 13) stored at 10 ± 2 °C and (**c**,**d**) 28 ± 2 °C; (**e**,**f**) composition B (MgO 47.5 wt%, MgCl_2_·6H_2_O 34.3 wt%, deionized water 18.2 wt%, MgO:MgCl_2_ molar ratio 7, H_2_O:MgCl_2_ molar ratio 12) stored at 10 ± 2 °C and (**g**,**h**) 28 ± 2 °C; (**i**,**j**) composition C (MgO 53.8 wt%, MgCl_2_·6H_2_O 30.2 wt%, deionized water 16.0 wt%, MgO:MgCl_2_ molar ratio 9, H_2_O:MgCl_2_ molar ratio 12) stored at 10 ± 2 °C and (**k**,**l**) 28 ± 2 °C [43].

Figure 5 illustrates the microstructural differences in MOC after 7 days of curing under different compositions and curing temperatures. High temperatures (28 °C) facilitate the formation of the 5·1·8 phase, whose crystals exhibit long needle-like shapes and interlaced growth, forming a dense microstructure. In contrast, at low temperatures (10 °C), the 3·1·8 phase is more prone to formation, with shorter crystals and a relatively loose structure. Additionally, with the increase in MgO content in the composition, more unreacted MgO remains even at high temperatures, indicating that the composition also has a certain influence on the formation of 3·1·8 and 5·1·8 phases. Due to its needle-like morphology, the 5·1·8 phase interweaves within the MOC matrix to form a network structure, thereby enhancing the mechanical properties of MOC cement [43].

**Figure 6:**
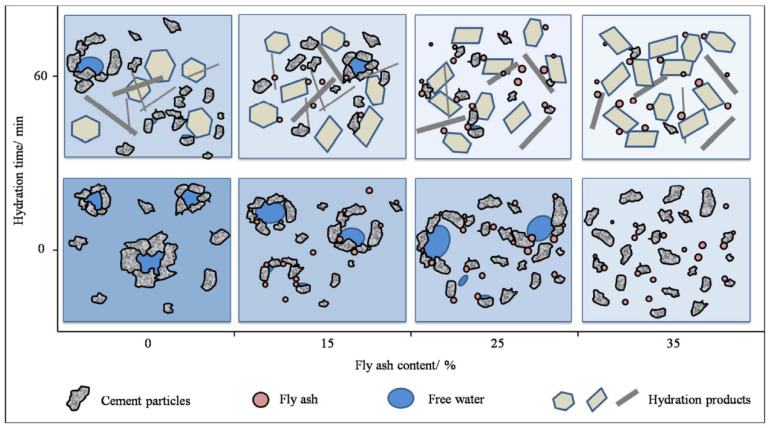
Effect of fly ash on cement particle dispersion at different hydration times [44].

When other active substances are incorporated into MOC, the types of hydration products generally remain unchanged. Wu et al. [44] investigated the influence of fly ash incorporation on the performance of MOC. Results show that in the early hydration stage (0 min), within the MOC paste without fly ash addition, cement particles aggregate into clusters under the action of van der Waals forces and Coulomb forces, leading to frequent flocculation phenomena. When the fly ash content reaches 35%, the morphological effect of its spherical particles promotes the independent dispersion of cement particles, releasing free water and thus improving the rheological properties of the paste. In the late hydration stage (60 min), the MOC paste generates more hydration products. Although fly ash does not directly participate in the hydration process, an increase in its content promotes the release of more free water, enabling cement particles to participate more fully in the hydration reaction and thereby increasing the quantity of hydration products. However, the presence of fly ash particles weakens the connections between hydration products and disrupts their network structure, leading to a decrease in mechanical properties, as shown in Figure 6.

### 4.2. Performance Characteristics of MOC

**Figure 7:**
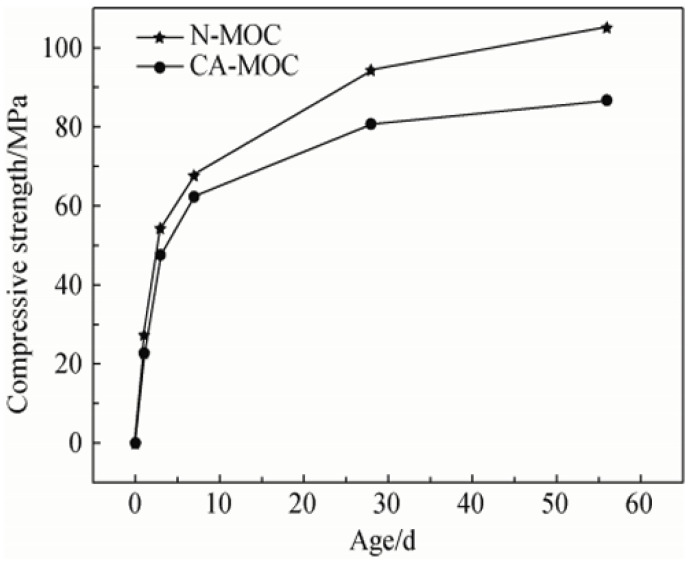
Compressive strength of magnesium oxychloride cement. N-MOC: unmodified MOC; CA-MOC: citric acid-modified MOC [46] (Reproduced with permission from [Xiaoyang Chen, Wanli Bi, Tingting Zhang], [The Modification of Magnesium Oxychloride Cement by Citric Acid]; published by [*The Chinese Ceramic Society*], [2019]).

MOC is a lightweight, rapid-setting and hardening magnesium-based cementitious material with low thermal conductivity and excellent resistance to both high and low temperatures [45]. As shown in Figure 7, in the study on the modification effect of citric acid (CA) on MOC, the compressive strength of CA-MOC was consistently lower than that of unmodified MOC at the same age. In addition, its 3-day compressive strength can reach approximately 60% of its 28-day strength, while its 7-day strength can exceed 70% of its 28-day strength [46]. This makes MOC suitable for construction in low-temperature conditions. According to Liang [47], when used as a binder for composite boards, MOC exhibits unique chemical compositions and structural features. Its hydration products possess a high melting point, superior thermal stability, and excellent fire resistance, ensuring structural integrity in high-temperature environments. These properties enable MOC to meet the performance requirements of low thermal conductivity, high flexural strength, good fire resistance, and strong interfacial bonding with wood boards.

Although MOC possesses numerous advantages, it also exhibits some limitations, such as insufficient water resistance and poor corrosion resistance. To address these issues, researchers have employed methods such as adding modifiers and applying coatings. Feng et al. [48] investigated the improvement of MOC’s water resistance by incorporating an optimal mixture of 1% phosphoric acid, 20% fly ash, and 1% calcium stearate or 1% styrene acrylic emulsion into MOC. The resulting samples achieved a 3-day compressive strength of 63.4 MPa and a 7-day strength of 75.1 MPa, with a softening coefficient as high as 0.9. This enhancement is primarily attributed to the coordination of [PO_4_]^3−^ ions with Mg^2+^ in the cement, which alters the hydrolysis ability of Mg^2+^ and the characteristics of its hydrolysis products. Consequently, this reduces the minimum Mg^2+^ concentration required for the formation of MOC hydrates, enabling the 5·1·8 phase to form even at low Mg^2+^ ion concentrations and improving its stability in water [49]. Fly ash, existing as spherical particles, acts as micro-aggregates, significantly reducing porosity and increasing structural density, thereby optimizing the overall structure [50]. Cao [51] conducted solution immersion tests on MOC-coated reinforced concrete (CRMOCC) to evaluate its durability. Electrochemical tests were used to analyze the corrosion of coated reinforcing bars, ultrasonic tests determined the crack development in CRMOCC, and mass loss was measured to assess degradation. Long-term durability was evaluated using parameters ω1, ω2, and ω3, and competitive failure analysis was performed to establish a durability degradation model. Li [52] studied the resistance of modified MOC to seawater corrosion. After 90 days of seawater immersion, the compressive strengths of MOC cement with 10% fly ash + 0.5% phosphoric acid, 10% slag + 0.5% phosphoric acid, and 8% silica gel + 0.5% phosphoric acid were 87.33 MPa, 78.13 MPa, and 97.80 MPa, respectively. The anti-corrosion coefficients for MOC with 20% fly ash + 0.5% phosphoric acid, 20% slag + 0.5% phosphoric acid, and 8% silica gel + 0.5% phosphoric acid were 0.75, 0.73, and 0.90, respectively. Zheng [53] found that MOC foam concrete prepared using animal and plant protein-based foaming agents exhibited better dimensional stability and lower tendency to collapse compared to samples made with chemical foaming agents. These samples also showed lower concentrations of dissolved Mg^2+^ and Cl^−^, leading to superior mechanical properties and water resistance.

### 4.3. Application in Soil Stabilization of MOC

**Figure 8:**
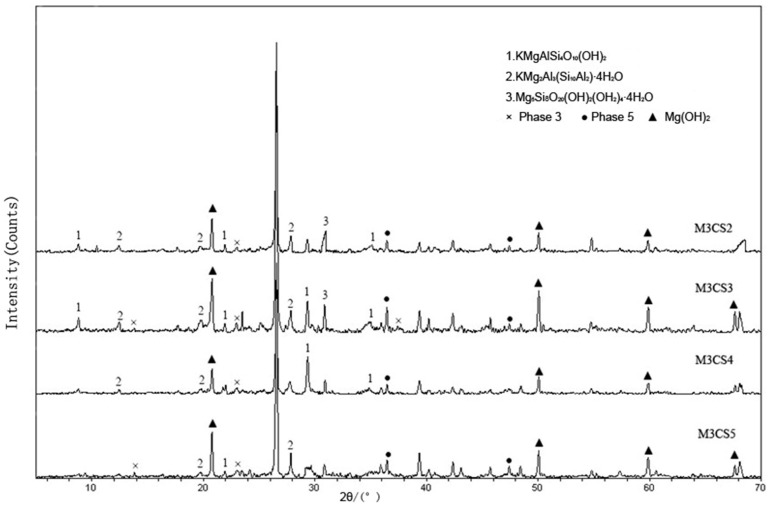
XRD patterns of solidified samples. M3 indicates a molar ratio of MgO/MgCl_2_ of 3:1; CS2, CS3, CS4, and CS5 represent mass ratios of MOC to chemical sludge of 3:100, 5:100, 10:100, and 20:100, respectively [54].

The curing process of soil by MOC is not instantaneous but occurs progressively over time. The fundamental mechanism behind this curing process lies in the hydration products of MOC, which physically and chemically bond the soil particles. Taking MOC-solidified dredged sludge as an example [54], after a period of curing, the XRD pattern (Figure 8) reveals the presence of the 5·1·8 phase, 3·1·8 phase, and Mg(OH)_2_. When MOC is dispersed in the dredged sludge, SiO_2_, Al_2_O_3_, or MgO within the sludge can react with unreacted Mg(OH)_2_ through hydration to form stable Mg-Si-Al hydrates (MgO·SiO_2_·Al_2_O_3_·nH_2_O), such as KMgAlSi_4_O_10_(OH)_2_ and KMg_2_Al_3_(Si_10_Al_2_)·4H_2_O. These hydration products create a crystalline network within the sludge, which not only enhances its mechanical strength but also immobilizes heavy metal ions, preventing their leaching.

**Figure 9:**
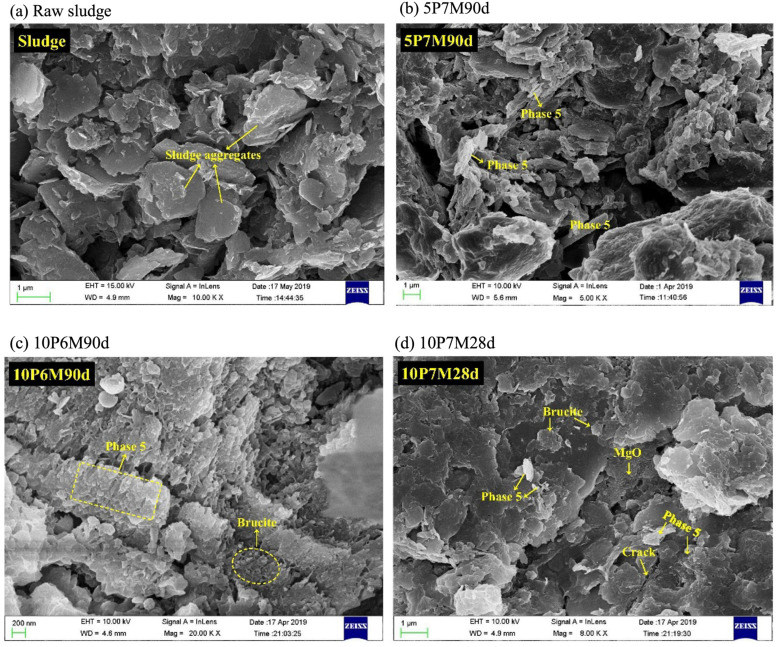

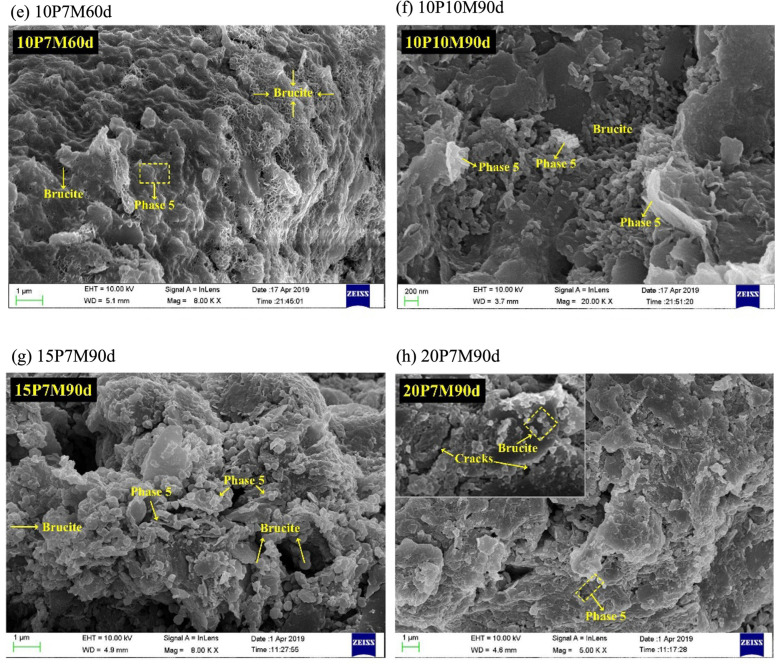
SEM images of MOC-solidified sludge. Sample (**a**): raw sludge; Sample (**b**): 5% MOC content, 7 MgO/MgCl_2_ molar ratio and 90 days curing time; Sample (**c**): 10% MOC content, 6 MgO/MgCl_2_ molar ratio and 90 days curing time. Sample (**d**): 10% MOC content, MgO/MgCl_2_ molar ratio of 7, curing time of 28 days; Sample (**e**): 10% MOC content, MgO/MgCl_2_ molar ratio of 7, curing time of 60 days; Sample (**f**): 1% MOC content, MgO/MgCl_2_ molar ratio of 10, curing time of 90 days; Sample (**g**): MOC content of 15%, MgO/MgCl_2_ molar ratio of 7, curing time of 90 days; Sample (**h**): MOC content of 20%, MgO/MgCl_2_ molar ratio of 7, curing time of 90 days [55].

After MOC curing of sludge-like soil, significant changes occur in its microstructure [55]. The original sludge exhibits a structure composed of large particles with substantial voids, as shown in Figure 9a. The addition of MOC renders the microstructure more compact and reduces porosity. A high MgO/MgCl_2_ molar ratio promotes the formation of the 5·1·8 phase and Brucite, but excessive Brucite induces microcracks; conversely, a low MgO/MgCl_2_ molar ratio restricts the formation of the 5·1·8 phase due to insufficient MgCl_2_, leading to crack occurrence, as shown in Figure 9b–h. In the sample cured for 60 days (Figure 9e), the needle-like and columnar 5·1·8 phase, as well as plate-like brucite Mg(OH)_2_ and other hydration products are observed. These hydration products not only fill the voids between particles, reducing the presence of large voids, but also interlock with each other and bind sludge particles/aggregates to form a dense network. In the sample cured for 90 days (Figure 9h), continuous Brucite formation causes volume expansion, which becomes the primary cause of strength degradation at the later stage. Consequently, this process refines the pore structure and enhances the compactness and strength of the cured sludge.

**Figure 10:**
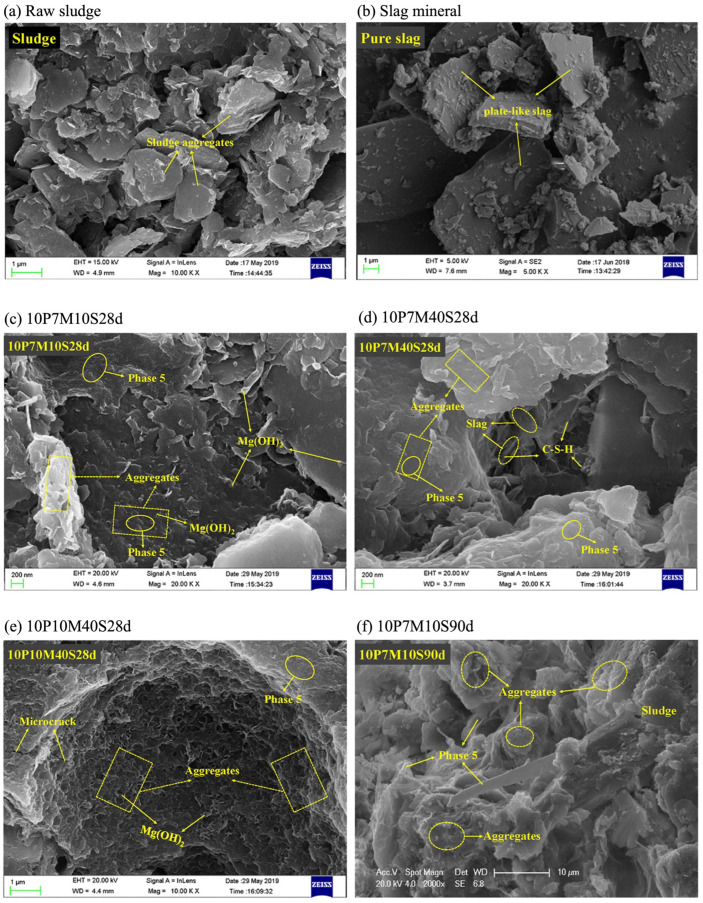

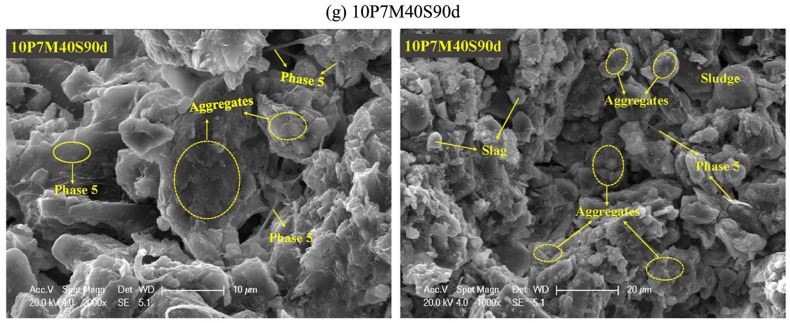
SEM images of slag-modified MOC-solidified sludge. Sample (**a**): raw sludge; Sample (**b**): slag mineral; Sample (**c**): MOC content of 10%, MgO/MgCl_2_ molar ratio of 7, GBFS content of 10%, age of 28 days; Sample (**d**): 10% MOC, 7 MgO/MgCl_2_ molar ratio, 40% GBFS, 28 days; Sample (**e**): indicates a MOC content of 10%, a MgO/MgCl_2_ molar ratio of 10, a GBFS content of 40%, and a conservation age of 28 days; Sample (**f**): indicates a sample with a MOC content of 10%, a MgO/MgCl_2_ molar ratio of 7, a GBFS content of 10%, and a curing age of 90 days; Sample (**g**): indicates a sample with a MOC content of 10%, a MgO/MgCl_2_ molar ratio of 7, a GBFS content of 40%, and a curing age of 90 days [56]. (Reproduced with permission from [Dongxing Wang, Xiangyun Gao, Xiqi Liu, Gang Zeng], [Strength, durability and microstructure of granulated blast furnace slag-modified magnesium oxychloride cement solidified waste sludge]; published by [Journal of Cleaner Production], [2025]).

Wang et al. [56] found that in their study of the microstructure of granulated blast furnace slag (GBFS)-modified MOC solidified waste sludge, non-solidified raw sludge exhibited a complex fabric characterized by large packets of disordered aggregates/particles and significant inter- and intra-aggregate pores. In contrast, the solidified sludge showed a marked reduction in large pores, resulting in a more compact matrix. Hydrated products, including brucite, floccular C-S-H gels, and polymerized-gel-like 5·1·8 phase, were observed, as illustrated in Figure 10.

Substantial experimental data have fully validated the remarkable solidification effect of MOC on gravel soil, sludge, and subgrade soil. As presented in Table 2, key physical properties, including density and compressive strength, for different soil types were systematically recorded before and after MOC-based solidification treatment. Through in-depth analysis and comparison of these data, it is clearly demonstrated that the performance of various soils has been significantly improved under the action of MOC.

## 5. Magnesium Oxysulfate Cementitious Material

### 5.1. Hydration Products of MOS

MOS is an air-hardening cementitious material produced by mixing and stirring reactive magnesium oxide with a magnesium sulfate heptahydrate solution [64]. When activated MgO reacts with the MgSO_4_ solution, leading to an ionization reaction that generates [Mg(OH)(H_2_O)_n_]^+^ and OH^−^. These ions form a hydrated film on the surface of MgO. Subsequently, OH^−^ reacts with [Mg(OH)(H_2_O)_n_]^+^ to form Mg(OH)_2_. This compound then further interacts with Mg^2+^, SO_4_^2−^ and OH^−^ in solution to form the alkaline magnesium sulfate complex salt [Mg_x_(OH)_y_·(SO_4_)_z_·nH_2_O].

**Figure 11:**
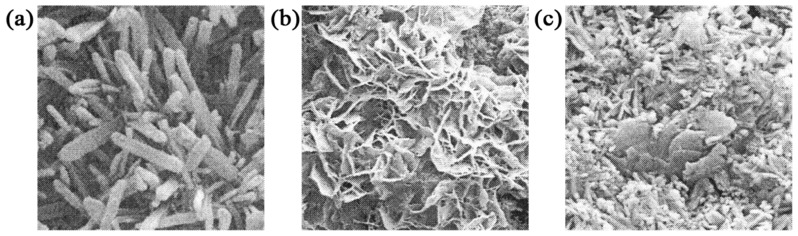
Hydration product of magnesium oxysulfate cement and the morphology of basic magnesium sulfate: (**a**) needle stick-shaped; (**b**) petal-shaped; (**c**) granular [1].

**Figure 12:**
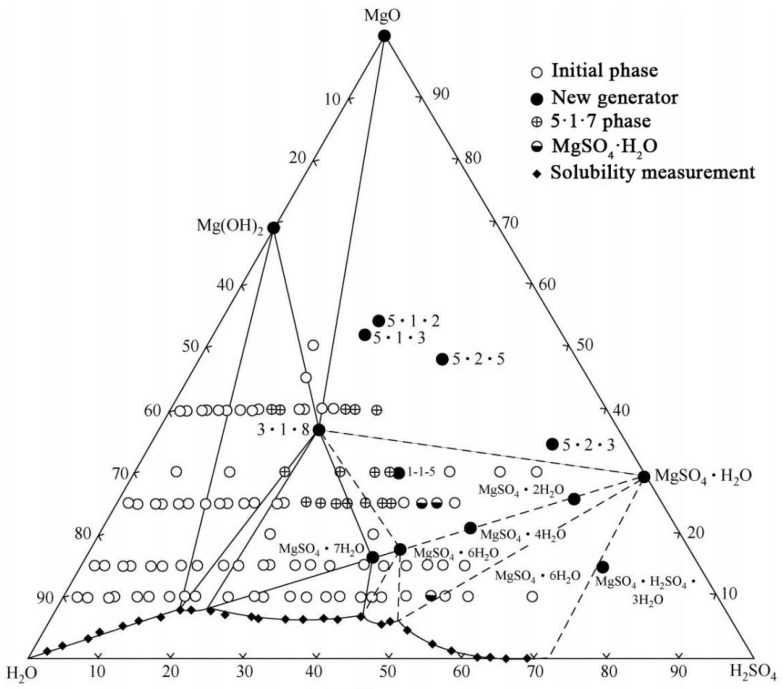
System of MgO-MgSO_4_-H_2_O at (23 ± 3) °C [66].

The common morphologies of MOS include the needle rod, petaloid, and granular forms, as illustrated in Figure 11. Cole [65] concluded that the primary mineral phases of MOS formulated in the MgO-MgSO_4_-H_2_O ternary system are the 5·1·3 phase, 3·1·8 phase, 1·2·3 phase, and 3·1·8 phase, with only the 3·1·8 phase being stable at 35 °C. Through differential thermal analysis, Raman spectroscopy, and X-ray powder diffraction, Dinnebier [13] discovered that the 3·1·8 phase exhibits sub-stability within the MgO-MgSO_4_-H_2_O ternary system at room temperature. Urwongse [66] examined the ternary phase diagram of the MgO-MgSO_4_-H_2_O system at room temperature (23° ± 3 °C) (Figure 12). The hydration product of MOS, the 3·1·8 phase, is challenging to form at room temperature, with its content not exceeding 50%. This limitation accounts for the lower strength and poorer water resistance of MOS compared to MOC. In their efforts to modify MOS cement, Yu et al. [67] succeeded in producing a new hydration product, the 5·1·7 phase, characterized by a needle-and-rod morphology, by incorporating chemical admixtures. This modification significantly enhances the strength and durability of MOS cement. The reaction formulas for the formation of the 5·1·7 and 3·1·8 phases are presented in Equations (3) and (4).

(3) $$5MgO+{Mg}^{2+}+{{SO}_{4}}^{2-}+12H_{2}O\to 5{Mg(OH)}_{2}\cdot {MgSO}_{4}\cdot 7H_{2}O$$

(4) $$3MgO+{Mg}^{2+}+{{SO}_{4}}^{2-}+11H_{2}O\to 3{Mg(OH)}_{2}\cdot {MgSO}_{4}\cdot 8H_{2}O$$

### 5.2. Performance Characteristics of MOS

Compared to MOC, the use of MgSO_4_ solution as a substitute for MgCl_2_ solution eliminates several drawbacks associated with moisture absorption, brine return, and corrosion of steel reinforcement. However, this substitution results in lower mechanical properties and diminished water resistance [68]. To enhance the performance of MgSO_4_ cement, researchers typically incorporate chemical admixtures or adjust the ratios of raw materials.

**Figure 13:**
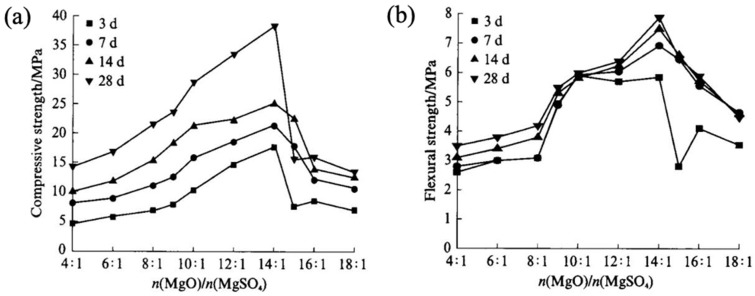
Effect of n(MgO)/n(MgSO_4_) on mechanical properties of MOS: (**a**) compressive strength; (**b**) flexural strength [71]. (Reproduced with permission from Ba, M.; Zhu, J.; Xue, T.; Liu], [Influence of Molar ratio on properties of magnesium oxysulfate cementitious materials]; published by Journal of Building Materials], [2018]).

In their study of the hydration behavior of MOS with citric acid as an admixture, Wu et al. [69] discovered that the addition of citric acid resulted in the formation of a stable organic magnesium complex layer on the surface of magnesium oxide. This layer inhibited the formation of Mg(OH)_2_ and promoted the generation of the 5·1·7 phase. When the content of active magnesium oxide in the modified magnesium oxysulfate cement was higher, its hydration exotherm, 28-day strength, and workability were significantly enhanced. Wang et al. [70] used citric acid (CA) and sodium alginate (SA) to synergistically modify magnesium oxysulfate cement. The results indicated that after modification with 1.2% CA and 0.4% SA, the internal structure of the cement formed a three-dimensional gel network, leading to pore filling and reduced water permeability. Consequently, the 28-day softening coefficient increased from 0.44 to 0.89, representing a 102.3% improvement. Ba et al. [71] highlighted that the molar ratios of n(MgO)/n(MgSO_4_) and n(H_2_O)/n(MgSO_4_) significantly influenced the performance of magnesium oxysulfate cement. Specifically, when n(H_2_O)/n(MgSO_4_) was fixed at 19:1, both the initial setting time and net slurry fluidity decreased as n(MgO)/n(MgSO_4_) increased. Additionally, flexural and compressive strengths declined when n(MgO)/n(MgSO_4_) exceeded 14:1. This is attributed to the increasing content of residual MgO and Mg(OH)_2_, which loosens the hardened structure and consequently reduces mechanical properties, as illustrated in Figure 13.

### 5.3. Application in Soil Stabilization of MOS

**Figure 14:**
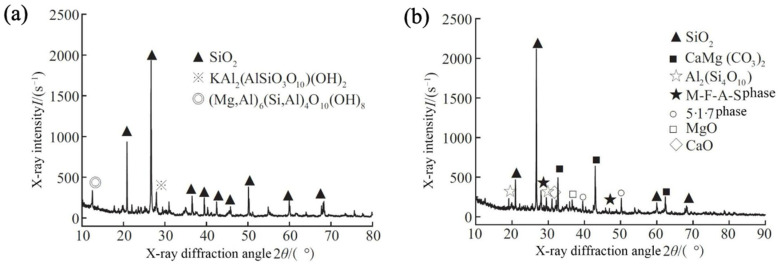
XRD patterns of mudflat soft clay and mudflat soft clay solidified by magnesium oxysulfate cement: (**a**) soft soil of tidal flats; (**b**) magnesium sulfide cement solidifies the soft soil of tidal flats [72].

The mechanism of MOS curing soil primarily involves hydrolysis and hydration reactions, ion exchange, as well as filling and carbonation effects. Throughout the curing process, MOS cement interacts with soil particles to produce new hydration products that effectively occupy the voids between these particles, thereby enhancing the bond strength among them. When utilizing MOS curing mudflat soft clay [72], the active MgO present in the modified MOS dissolves into Mg^2+^ and OH^−^ ions within a MgSO_4_ solution. When the concentrations of Mg^2+^, SO_4_^2−^, and OH^−^ reach a specific threshold, they directly form the 5·1·7 phase, resulting in a certain degree of strength that tightly binds soil particles together to create a spatial mesh structure, as illustrated in Figure 14. Furthermore, Al_2_O_3_, Fe_2_O_3_, and active SiO_2_ from the chemical admixture react with the MgO-MgSO_4_-H_2_O ternary system of the modified MOS to produce an M-F-A-S gel phase; this reaction is represented by Equation (5). The gel fills the crystal lattice of the 5·1·7 phase, leading to a dense and compact structure that enhances water resistance in MOS-cured beach soft soil.

(5) $${MgSO}_{4}+MgO+{Fe}_{2}O_{3}+{Al}_{2}O_{3}+SiO_{2}+H_{2}O\to M-F-A-S$$

**Figure 15:**
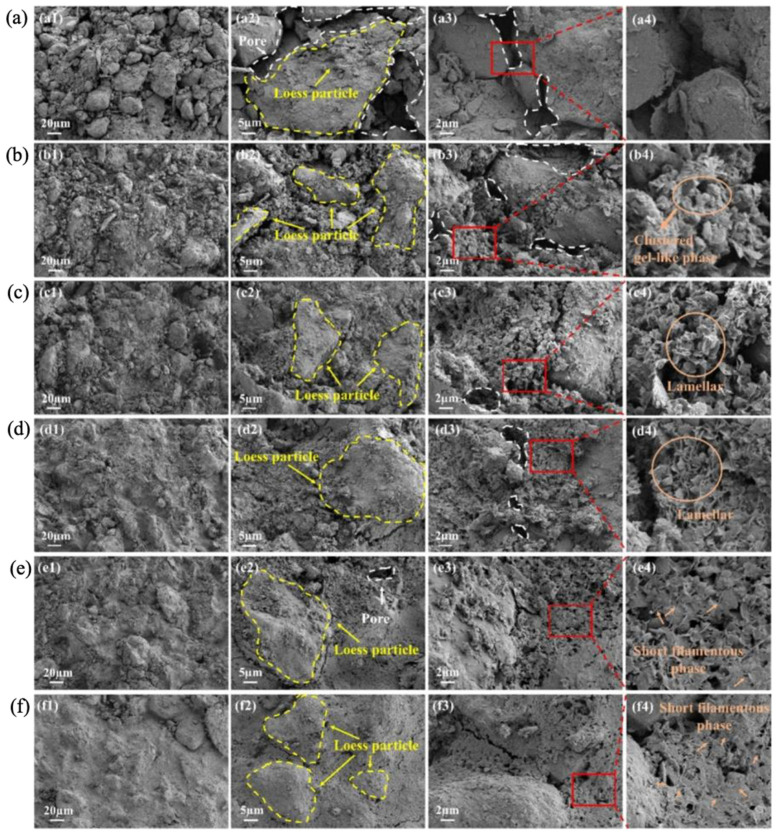
SEM micrographs of the pure loess (**a**) and solidified loess samples with different MOS binders: (**b**) 5%, (**c**) 7%, (**d**) 9%, (**e**) 11%, and (**f**) 13%, Subfigures numbered 1, 2, and 3 represent different magnification levels, subfigure numbered 4 shows a magnified view of subfigure 3 [73].

**Figure 16:**
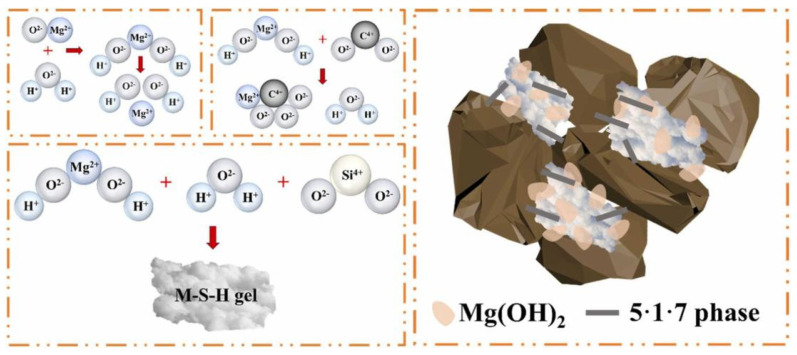
Schematic diagram of curing mechanism [74].

After the addition of MOS to the soils, the microstructure of the soils will change considerably, and most obviously, the pore structure will become denser. Figure 15 shows the microstructures of pure loess as well as MOS-cured loess samples [73]. The pores were filled by a large number of clustered gel-like substances, flakes, and a small amount of short filaments. Yan et al. [74] investigated the synergistic curing of loess by MOS with coal gangue. It was concluded that MOS reacted with components in loess to produce M-S-H, Mg(OH)_2_ and a small amount of 5·1·7 phase, and these hydration products filled the voids between soil particles and made the soil structure more compact. In addition, the layered hydration products of Mg(OH)_2_ and Ca(OH)_2_ formed a network structure in the soil, which enhanced the compaction and mechanical properties of the soil. Part of Mg(OH)_2_ reacted with CO_2_ to form MgCO_3_, which was attached to the unhydrolyzed hydration products in the form of cohesive particles, as shown in Figure 16.

MOS has a good solidification effect on soil, which is mainly reflected in the improvement of the compressive strength of the solidified soil and the simultaneous enhancement of its water resistance. In recent years, the research on soil solidified by MOS is shown in Table 3.

## 6. Magnesium Phosphate Cementitious Material

### 6.1. Hydration Products of MPC

**Figure 17:**
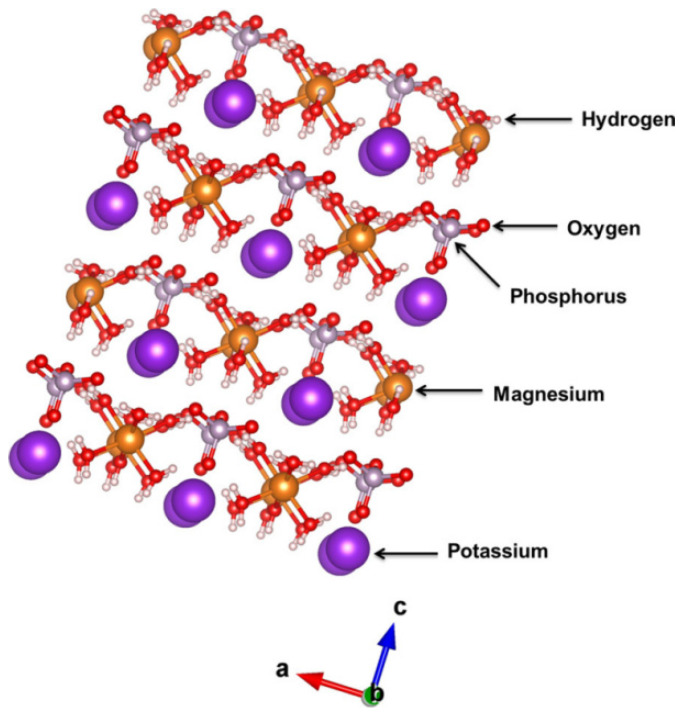
Crystal structure diagram of bird guano stone struvite [14].

MPC is mainly formulated from dead burned magnesium oxide, phosphate and retarder and other mineral admixtures in certain proportion, which is a cementitious system different from ordinary cement. Its hydration product is MgNH_4_PO_4_-6H_2_O (struvite) [78,79] generated by acid-based chemical reaction between MgO and soluble phosphate, and its crystal structure is shown in Figure 17.

**Figure 18:**
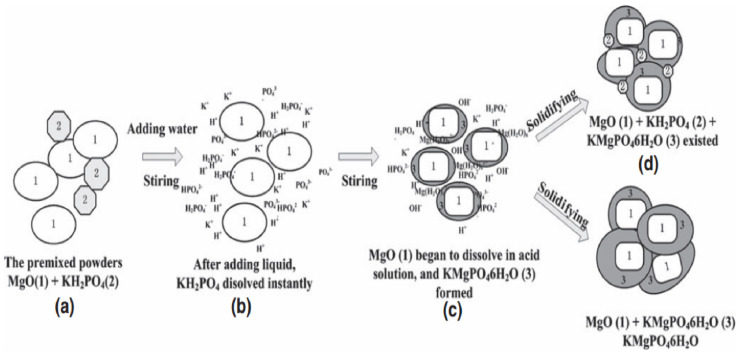
Hydration process and hydration products of MKPC [82].

Differences in conditions such as phosphorus to magnesium ratio, retardant, and hydrogel ratio may lead to different hydration products of MPC. In the course of thermal analysis of MPC, Abdelrazig [80] found that the main hydration product of MPC is MgNH_4_PO_4_·6H_2_O, which is Mg(NH_4_)_2_(HPO_4_)_2_·4H_2_O in the early stage of the reaction, and is produced only after the reaction with H_2_O, while the hydration products may also have Mg_2_P_2_O_7_ and Mg_3_(PO_4_)_2_. Sugama [81] suggested that the hydration products of MPC also have Mg(OH)_2_. Xu et al. [82] found that Mg_2_K(HPO_4_)_2_·15H_2_O, Mg_3_(PO_4_)_2_·22H_2_O, MgHPO_4_·7H_2_O and MgKPO_4_·H_2_O are the intermediate transition phases of magnesium potassium phosphate (MKPC) in their study on the characterization method of MKPC. The main hydration products are MgKPO_4_·6H_2_O and gel, and the hydration process is shown in Figure 18. In general, MgNH_4_PO_4_·6H_2_O as the main hydration product of MPC is unanimously agreed by many scholars, which is the most abundant and has the best bonding performance, affecting the environmental adaptability, stability, curing effect and many other properties of MPC.

### 6.2. Performance Characteristics of MPC

**Figure 19:**
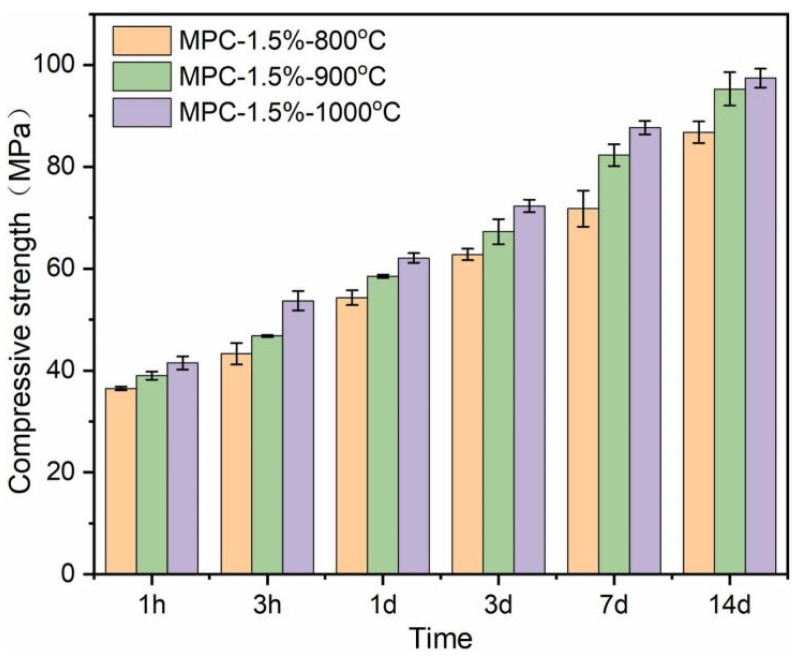
Influence of different calcination temperatures on compressive strength of MPC [87]. (Reproduced with permission from [Chen, X.; Guo, J.; Yu, H.; Zhang, G.; Kang, Y.; Zhang, M.; Hao, T.; Du, Q], [mpact of calcination temperatures on lithium magnesium slag for enhanced magnesium phosphate cement properties]; published by Construction and Building Materials], [2024]).

Compared with traditional Portland cement, MPC is characterized by fast hydration, rapid setting and hardening, high early strength, good adhesion, abrasion resistance, and compatibility [83]. Portland cement doped with blast furnace slag loses at at least 84% of its strength after 4 h of calcination at 900 °C due to evaporation of water and pressure buildup in the pores, while composite MPC doped with 5% fly ash and 15% silica fume has the best high-temperature resistance [84,85]. The dead burned MgO used for MPC is mainly the sintered MgO obtained by calcining magnesite at 1500~1700 °C. The MgO obtained in this way has a complete crystallization and dense structure, and its reactivity is low, and a relatively long coagulation time can be obtained with a small dosage of retardant [86]. It can be found from the study by Chen [87] on the effects of different section firing temperatures on the properties of MPC that MPC has the characteristic of high early strength, the compressive strength of 1 h can reach about 40 MPa, and the compressive strength of 14 d can reach more than 80 MPa. With the increase in calcination temperature, the increase in late strength gradually decreases, but the overall strength still shows an increasing trend, as shown in Figure 19.

**Figure 20:**
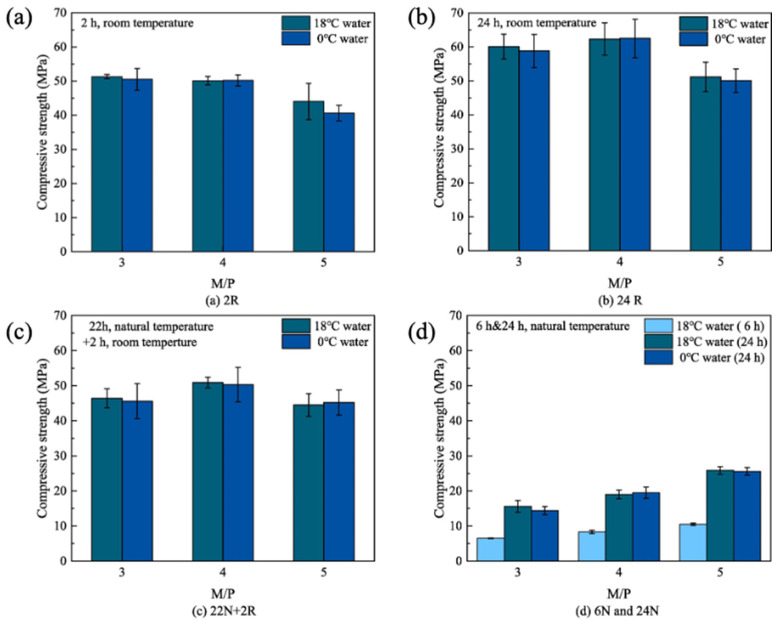
Compressive strength of MPC pastes with different M/P ratios under different curing modes. (**a**) 2R: 2 h room curing (20 °C). (**b**) 24R: 24 h room curing (20 °C). (**c**) 22N+2R: 22 h natural curing (−27 °C~17 °C) followed by 2 h room curing (20 °C). (**d**) 6N and 24N: 6 h and 24 h natural curing (−27 °C~17 °C) [91]. (Reproduced with permission from [Jie Yuan, Xin Huang, Xin Chen, Qian Ge, Zhichao Zhang], [Early-age mechanical properties and hydration degrees of magnesium phosphate cement paste in freezing winter of cold regions]; published by Elsevier], [2022]).

Similarly to the other two magnesium cementitious materials, MPC has poor water resistance. Since the acidic medium formed by phosphate in water dissolves the hydration products of magnesium phosphate cement, unreacted phosphate is easily leached out by water, leading to a decrease in strength and an increase in porosity, thus affecting water resistance. Su [88] found that the silica–aluminum minerals in sintered silt, in which reactive Al_2_O_3_ can participate in the reaction to form an aluminum-containing gel phase, have the effect of blocking pores, making it difficult for water to flow. At the same time, the sintered silt itself can be used as a filler to fill in the pores inside the MPC cement matrix, indirectly increasing the densification and improving the impermeability. Using citric acid to enhance the water resistance of MPC, Sun et al. [89] found that citric acid did not affect the hydration products of MPC but promoted the full reaction between H^+^ and MgO and formed a chelate film during the hydration process, thus improving the water resistance of MPC. For the repair of broken concrete structures in alpine regions, Chen et al. [90] investigated the effects of molding temperature and magnesium phosphorus mass ratio on the frost resistance of MPC mortar at high altitude. When considering the environmental and material factors, it was observed that the increase in altitude, the decrease in temperature, and the decrease in the water-cement ratio (M:P) resulted in the improvement of the frost resistance of MPC mortars. Further analysis of the bubble structure and water absorption properties revealed that MPC mortars with higher bubble content, smaller bubble spacing and lower water saturation had better frost resistance. After freeze–thaw cycles, the time required for the mass of MPC mortar to decrease to 95% of its initial mass was shorter than the time required for its dynamic elastic modulus to decrease to 60% of its initial value. Yuan et al. [91] found that the early strength of MPC increased with the increase in the M/P ratio under severe cold conditions at high latitudes. When the magnesium-phosphorus mass ratios reached 4 and 5, the 24-d strength could reach 50 MPa and 25 MPa, respectively, as shown in Figure 20. This phenomenon is explained by the fact that MPC pastes with higher M/P ratios generate more heat of hydration. This heat generated by the mixing of water and hydration reactions slows down the cooling of the sample, thus providing the necessary energy for the chemical reactions and preventing premature freezing of the pore solution.

### 6.3. Application in Soil Stabilization of MPC

MPC solidified soil relies mainly on chemical bonding, adsorption, and physical encapsulation, which can effectively solidify and stabilize heavy metal ions, thus reducing their mobility and leachability. When MPC cures zinc-contaminated soils, its hydration product MgKPO_4_∙6H_2_O (K-type struvite) is capable of curing heavy metals Zn^2+^ by forming insoluble salts precipitated adsorbed or chemically bound to them, such as Zn_3_(PO_4_)_2_∙4H_2_O, Zn(OH)_2_, CaZn_2_(PO_4_)_2_∙2H_2_O. In low pH environments such as acid rain, these hydration products may undergo a reversal reaction and gradually transform into the stable phases Mg_3_(PO_4_)_2_·22H_2_O, Mg_3_(PO_4_)_2_·8H_2_O, which leads to the dissolution of Zn compounds adsorbed or precipitated on them [92].

Wang et al. [93] suggested that MPC adsorbs and stabilizes heavy metals in soil by building a stable crystalline network. With the fluctuation of water content, MgKPO_4_·6H_2_O can be transformed into other more stable mineral forms, such as Mg_3_(PO_4_)_2_·22H_2_O, which can further enhance the curing effect. However, the curing effect can be affected by the concentration of heavy metals in the soil and the water-solid ratio. At a water-solid ratio (W/S) of 0.50, the content of mobile Pb^+^ in the soil rises, resulting in more Pb^+^ leaching from the MPC-cured soil. As the W/S ratio increased, the concentration of Pb^+^ in the leachate increased, while the pH showed a decreasing trend. This phenomenon may be attributed to the generation of more Mg_3_(PO_4_)_2_·8H_2_O under higher moisture conditions, leading to a decrease in the amount of MgKPO_4_·6H_2_O, which in turn increased the leaching of Pb^+^.

**Figure 21:**
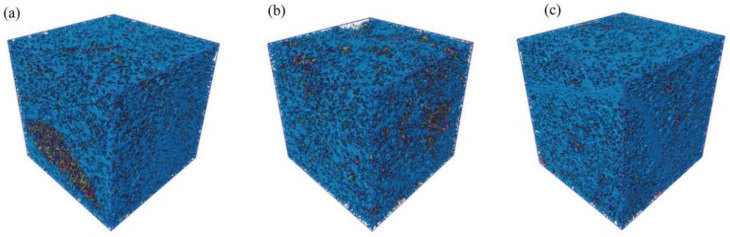
Total pores in real morphology of three samples: (**a**) 0%, (**b**) 6%, (**c**) 15% [94].

**Figure 22:**
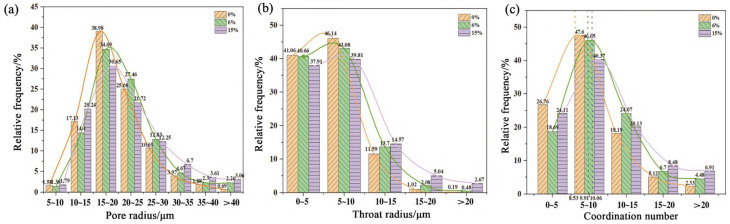
The distribution of equivalent pores, throat size, and coordination number of three samples: (**a**) equivalent pore size, (**b**) throat size, and (**c**) coordination number [94]. (Reproduced with permission from [Feng, Y.-S.; Du, Y.-J.; Reddy, K.R.; Xia, W.-Y], [Performance of two novel binders to stabilize field soil with zinc and chloride: Mechanical properties, leachability and mechanisms assessment.]; published by *Constr Build Mater*], [2018]).

Figure 21 and Figure 22 show the changes in pore structure of the Yellow River silt deposited low liquid limit chalky soil reinforced by MPC with different dosages, as studied by Fan et al. [94]. With the increase in MPC doping, more hydration product MgNH_4_PO_4_·6H_2_O was observed to bond the soil particles tightly. At the same time, the excess MgO filled in the interstices of soil particles and built up the interaction structure between hydration products and soil particles, which enhanced the bonding force between soil particles and reduced the number of small pores. Although the total volume of the cured soil remained constant, the aggregation of soil particles left specific pore spaces, leading to an increase in the number of large pores. This not only enhanced the connectivity between the pores, but also the hydration products of MPC showed excellent volume stability. Due to the cementation of MgNH_4_PO_4_·6H_2_O with soil particles, the volume of the formed soil skeleton was less affected by hydration. These two factors together resulted in an increase in the porosity of the cured soil.

**Figure 23:**
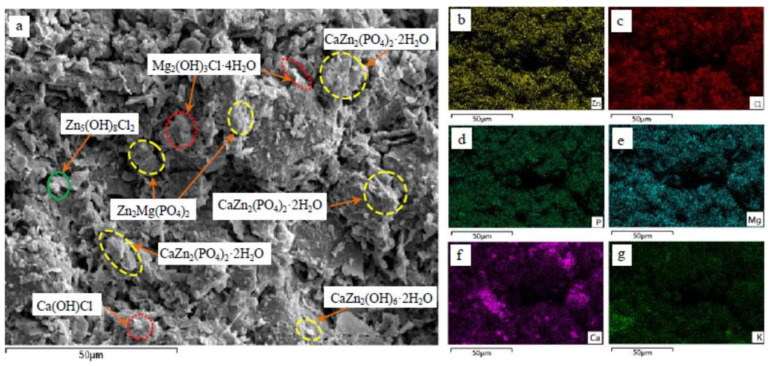
SEM-EDS image of the KMP stabilized soil and the element distribution maps: (**a**) SEM image; (**b**) distribution map of Zn; (**c**) distribution map of Cl; (**d**) distribution map of P; (**e**) distribution map of Mg; (**f**) distribution map of Ca; and (**g**) distribution map of K [95]. (Reproduced with permission from [Ya-Song Feng, Yan-Jun Du, Krishna R. Reddy, Wei-Yi Xia], [Performance of two novel binders to stabilize field soil with zinc and chloride: Mechanical properties, leachability and mechanisms assessment.]; published by Elsevier], [2025]).

The stabilizing effect of reactive magnesia activated blast furnace slag-based binder (GM) and phosphate-based binder (KMP) on soils containing mixed pollutants of zinc and chlorine was investigated by Feng et al. [95] who found that the acid-base reaction products of the KMP curing agent, MgKPO_4_·6H_2_O and Mg_3_(PO_4_)_2_·8H_2_O, possessed excellent chemical bond strengths, and were able to fill the soil pores effectively and Zn and Cl binding products such as Zn_3_(PO_4_)_2_·4H_2_O, Zn(OH)_2_ and CaZn_2_(PO_4_)_2_·2H_2_O were also detected in the KMP-cured soil, which were able to effectively immobilize Zn and Cl in the soil matrix, thus reducing their leaching concentrations, as shown in Figure 23.

MPC has demonstrated good curing effects on contaminated soil, effectively reducing the leaching of pollutants and improving soil stability. Table 4 presents the progress of MPC in curing different types of soils.

## 7. Research Status of Other MBCMs in Soil Solidification

**Figure 24:**
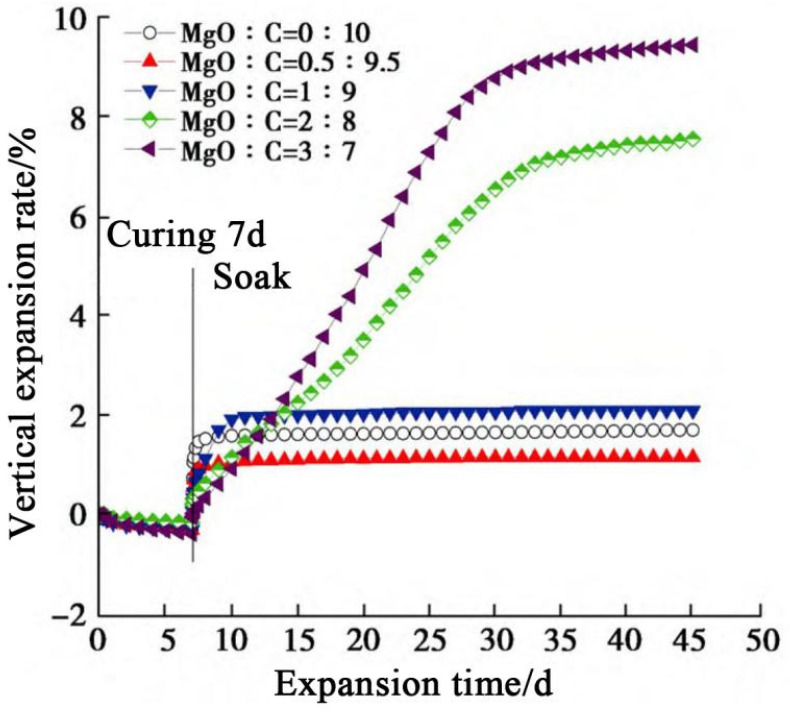
MgO vertical expansion rate of cement-stabilized soil [102].(Reproduced with permission from [Li, W.; Sun, Z.; Zhuang, Y.; Xiao, H.; Fu, Z.; Zhou, X.], [Mechanical Properties, Swelling Behavior, and Microscopic Mechanisms of Sulfate-Contaminated Soil Stabilized by Magnesium Oxide Composite Cement.]; published by the Chinese Journal of Geotechnical Engineering], [2024]).

In the research domain of soil stabilization using magnesium-based cement, numerous scholars have conducted extensive and in-depth investigations. Cai [101] employed MgO and CO_2_ as novel curing agents to replace traditional Portland cement for the reinforcement of weak soils. Figure 24 illustrates that the specific gravity of soil treated with MgO decreased relative to untreated soil, while the specific gravity of carbonized soil exhibited a slight reduction compared to its pre-carbonization state. The specific gravity of the soil was found to decrease with higher MgO content, increased reactivity of magnesium oxide, or a lower water-cement ratio. The porosity and saturation of MgO-treated soil significantly decreased compared to pre-carbonization levels and exhibited a power function increase with the rising water-ash ratio. Conversely, the porosity and saturation of carbonized soil decreased linearly with an increasing MgO activity index, while they increased with an increasing soil liquid limit. Additionally, the porosity and saturation were found to increase with a larger water-soil ratio (w_o_/w_l_).

Given that conventional cement curing can lead to swelling, loss of strength, and reduced durability in sulfate soils, Li [102] and colleagues explored the use of magnesium oxide as a partial substitute for cement in the treatment of sulfate-bearing soils (gypseous soil). Their experimental findings revealed that when the MgO-to-cement ratio was set at 0.5:9.5, the expansion of the treated soil was minimized to 1.15%, which is 0.55% lower than that observed in soil treated solely with cement. This approach effectively inhibited the formation of ettringite. Furthermore, the subsequent formation of M-S-H was found to suppress the development of C-S-H, thereby diminishing the influence of C-S-H, as illustrated in Figure 24.

Yuan et al. [103] conducted a comprehensive study on the remediation of heavy metal-contaminated soils using hydrothermal carbon-modified magnesium silicate (MS-C). Their findings revealed that the incorporation of MS-C significantly altered the physicochemical properties of the soil samples. Specifically, the pH levels of the treated soils increased by 0.936 to 2.17 units, while the water-soluble organic carbon (WSOC) content was enhanced by approximately 10%. Furthermore, the bioavailability and toxicity of the heavy metals were substantially reduced, with decreases ranging from 20% to 86.7% after 60 days and from 26.6% to 73.2% thereafter. These results suggest that MS-C serves as an effective stabilizing agent for the remediation of both mono- and polymetallic contaminated soils.

## 8. Environmental Impacts of Soil Curing with MBCMs

The environmental impact of magnesium-based cementitious materials during the soil curing process is predominantly positive. In terms of energy consumption during the production process, the calcination temperature required for Portland cement clinker is significantly higher, ranging from 1300 to 1450 °C. In contrast, magnesium cement materials only require a calcination temperature of 750 to 850 °C. This substantially lower temperature requirement results in a significant reduction in energy consumption. Furthermore, the production process of magnesium cement is relatively straightforward, involving only one grinding and one calcination step. This contrasts with the more energy-intensive two grinding and one calcination process used for silicate cement, thereby conserving both energy and resources [9,104].

**Figure 25:**
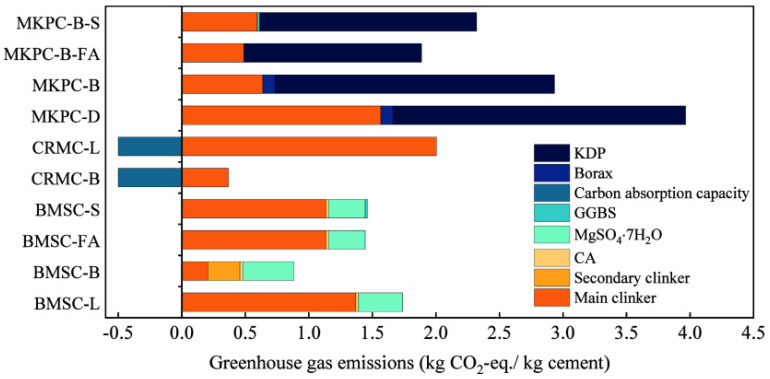
CO_2_ emissions of different types of magnesia-based cement. KDP: potassium dihydrogen phosphate. GGBS: ground granulated blast-furnace slag. CA: ctric acid. L-MgO: light-burned magnesium oxide produced from magnesite. D-MgO: dead-burned magnesium oxide produced from magnesite. LB-MgO: light-burned boron-containing magnesium oxide produced from salt lake magnesium residue. DB-MgO: dead-burned boron-containing magnesium oxide produced from salt lake magnesium residue. MKPC-B-S: magnesium potassium phosphate cement using DB-MgO and GGBS. MKPC-B-FA: magnesium potassium phosphate cement using DB-MgO and FA. MKPC-B: magnesium potassium phosphate cement using DB-MgO. MKPC-D: magnesium potassium phosphate cement using D-MgO. CRMC-L: carbonation reactive magnesium cement using L-MgO. CRMC-B: carbonation reactive magnesium cement using LB-MgO. BMSC-S: basic magnesium sulfate cement using GGBS. BMSC-FA: basic magnesium sulfate cement using FA. BMSC-B: basic magnesium sulfate cement using LB-MgO. BMSC-L: basic magnesium sulfate cement using L-MgO [105].

**Figure 26:**
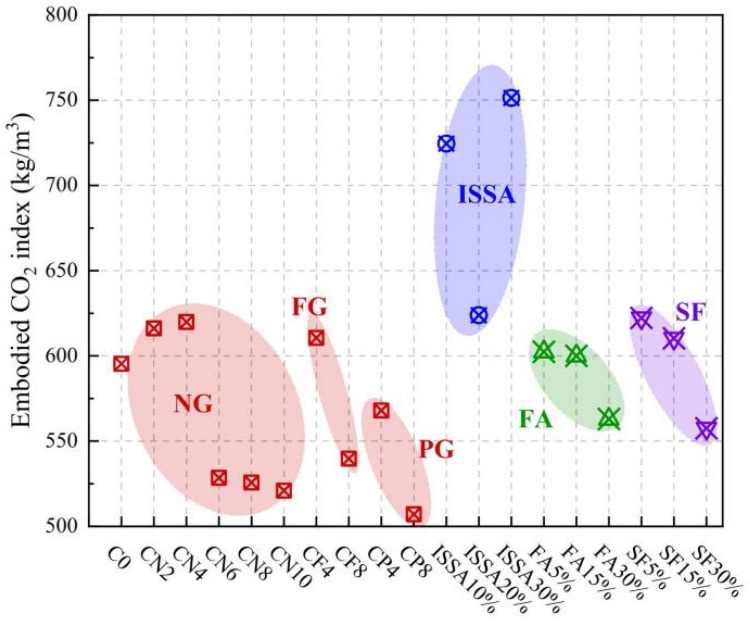
Evaluation and comparison of sustainability of MOCP. NG: natural gypsum; FG: flue gas desulfurization gypsum; PG: phosphogypsum; ISSA: incinerated sewage sludge ash; FA: fly ash; SF: silica fume [109]. (Reproduced with permission from [Cong Ma, Gege Chen, Jinyan Shi, Haijun Zhou, Weixin.

**Figure 27:**
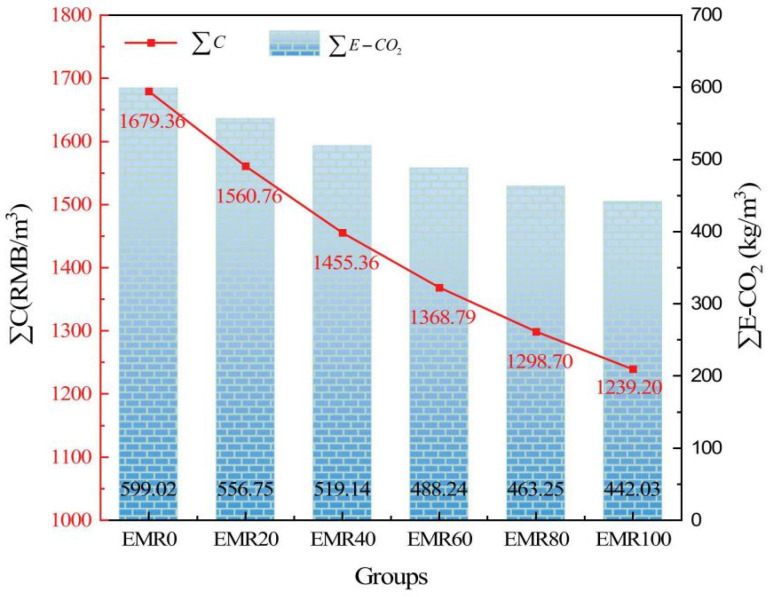
Total cost and total carbon emissions per cubic meter of specimens with different EMR dosages [110]. (Reproduced with permission from [Jilin Wang, Kaiyong Ma, SiKai Shang, Junjie Ran, HaomiaoXia, Ziyi Luo, Yingjie Li, Yingying Shi, Gege Chen, Zhuo Tang, Rongzhen Dong], [Exploration of the effects of electrolytic manganese residue on the environmental, economic, and engineering performance of magnesium oxychloride cement: A possible utilization method of electrolytic manganese residue], published by Elsevier [2024]).

Regarding specific carbon emission data, Figure 25 illustrates the carbon footprint associated with magnesium-based cements. Notably, MgO derived from magnesium residues in salt lakes, such as LB-MgO and DB-MgO, exhibits a significant reduction in CO_2_ emissions compared to MgO produced via conventional methods, such as L-MgO and D-MgO. The reductions are quantified as 81.66% and 60.33% for CO_2_-eq/kg, respectively [105]. Additionally, Tan et al. [106,107] achieved a reduction in carbon emissions by 41.47% and 52.44% through the incorporation of two supplementary cementitious materials, fly ash and blast furnace slag, into MKPC. This enhancement significantly improved the environmental sustainability of magnesium-based cements. LisKa et al. [108] employed X-ray diffraction analysis and scanning electron microscopy to investigate the carbonation of magnesium cement blocks. Following quantitative analysis using these techniques, it was determined that magnesium-based cements can achieve a carbonation level of at least 71%, thereby reducing total CO_2_ emissions to a maximum of 0.32 t/t. In contrast, Portland cements exhibit a maximum carbonation of 30% with CO_2_ emissions of 0.37 t/t. Ma et al. [109] investigated the influence of different admixtures on the carbon emissions per unit volume of magnesium oxychloride cement paste (MOCP) and evaluated their specific environmental impacts. The results showed that MOCP samples incorporating high-proportion gypsum, such as 80% flue gas desulfurization gypsum (FG) and 80% phosphogypsum (PG), exhibited significantly lower carbon emissions compared to those using traditional supplementary cementitious materials, including fly ash (FA), silica fume (SF), and incinerated sewage sludge ash (ISSA). As illustrated in Figure 26, the carbon emissions of samples with 80% FG and 80% PG decreased to approximately 500–550 kg/m^3^, while that of the traditional formula (e.g., 30% ISSA) was as high as over 750 kg/m^3^. Moreover, as a cementitious material, lightly calcined magnesia has a net carbon emission 40–50% lower than that of ordinary Portland cement (OPC), demonstrating remarkable environmental advantages. Wang et al. [110] conducted an in-depth study on the carbon emissions of MOC with different dosages of electrolytic manganese residue (EMR). The results indicated that the carbon emissions of the specimens showed a decreasing trend with the increase in EMR content. Specifically, compared with the reference group (0% EMR), the carbon emissions of samples with 20%, 40%, 60%, 80%, and 100% EMR were reduced by 7.06%, 13.34%, 18.49%, 22.67%, and 26.21%, respectively, as shown in Figure 27.

Ren, Yanliang Du], [Improvement mechanism of water resistance and volume stability of magnesium oxychloride cement: A comparison study on the influences of various gypsum], published by Elsevier [2022]).

However, the production process of magnesium-based cements is not without its drawbacks. Notably, the manufacturing of magnesium potassium phosphate cement is characterized by high energy consumption, particularly in the production of dead-burned MgO and potassium dihydrogen phosphate (KDP). Specifically, the production of each ton of KDP requires approximately 1.2 tons of coal and 200 kWh of electricity. This inevitably results in substantial consumption of fossil fuels, thereby exacerbating the risk of fossil fuel depletion [111].

Overall, the environmental impact of magnesium-based cements during the soil curing process exhibits dual characteristics. On the positive side, it not only contributes to the reduction in greenhouse gas emissions but also facilitates the recycling of resources. It excels in the treatment of industrial by-products and waste, thereby effectively alleviating environmental pressures and providing robust support for the sustainable utilization of resources. Additionally, its curing process is capable of absorbing significant amounts of CO_2_, offering the potential for achieving net-zero emissions. This capability holds strategic importance for the global response to climate change. On the negative side, the environmental burden resulting from energy consumption during the production process cannot be overlooked. However, through continuous technological innovation and process optimization, it is anticipated that these adverse effects will be further mitigated. This will enhance the environmental sustainability of magnesium-based cements, enabling it to play a more significant role in the field of soil curing and contribute more effectively to the balanced development between environmental protection and engineering construction.

## 9. Issues and Challenges

Magnesium-based cementitious materials offer a promising avenue for soil curing, presenting several advantages over traditional silicate cement, such as high early strength, reduced energy consumption, decreased pollution, and enhanced soil curing effects. However, the practical application of magnesium-based cementitious materials in soil curing is currently hindered by a series of critical challenges that impede their full potential and widespread utilization.

Foremost among these challenges is the insufficient stability of the hydration products of magnesium-based cementitious materials. The curing mechanism involving their interaction with soil particles remains inadequately understood. This lack of clarity results in variability of material properties, complicating the precise control of curing effects in actual engineering applications and posing significant risks to their large-scale and reliable deployment. Consequently, a thorough investigation into the hydration processes of magnesium-based cementitious materials and the microscale mechanisms of soil curing is essential for addressing the issue of unstable performance. This necessitates the application of advanced material analysis techniques to examine the formation and evolution of hydration products at the molecular and atomic levels, as well as their interactions with soil particles, including both chemical and physical bonding. Such research will provide a robust theoretical foundation for optimizing material formulations and curing processes.

Secondly, the composition of magnesium-based cement used as curing agents is highly complex and variable, encompassing a wide range of constituents. The dosage of different curing agents varies significantly, and the ratio of key components such as magnesium oxide to magnesium chloride, magnesium sulfate, and dihydrogen phosphate plays a pivotal role in determining the properties of the hydration products, microstructure, and macro-mechanical characteristics of magnesium-based cementitious materials. This, in turn, influences the various curing mechanisms at play. While numerous studies have documented the application of magnesium-based cementitious materials, there is a notable absence of in-depth analysis regarding their curing mechanisms. This gap has led to considerable divergence in the understanding of the principles underlying the same curing agent and the curing process within both academic and engineering communities, hindering the development of a unified and effective application guideline. Consequently, it is imperative to enhance systematic research into the curing mechanisms of various curing agents. By employing advanced microscopic testing techniques such as X-ray diffraction, scanning electron microscopy, and energy dispersive spectroscopy, a detailed investigation of the interactions between soil, soil moisture, and curing agents at the microscopic level can be conducted. This includes examining dynamic behaviors such as ion exchange, dissolution and precipitation, and crystallization growth. Subsequently, the development of a comprehensive curing mechanism model will provide a solid scientific basis for the rational selection and accurate application of curing agents, thereby enhancing the reliability and stability of the cured soil.

Furthermore, the composition and characteristics of natural soil are exceedingly complex. The particle size distribution, mineral composition, and chemical content vary significantly due to the diverse environments in which soils form. Additionally, soil texture is subject to continuous alteration by natural processes such as wind erosion, water erosion, and freeze–thaw cycles. These factors interact in complex ways, leading to substantial variations in the curing effects of magnesium-based cementitious materials curing agents under different soil conditions. This variability in curing effectiveness not only restricts the application scope of magnesium-based cementitious materials in soil curing projects but also may result in increased project costs and compromised quality stability. To address this challenge, it is essential to conduct a comprehensive and in-depth investigation to systematically evaluate the applicability of various types of magnesium-based cementitious materials curing agents to different soil types. By conducting extensive laboratory tests and field application case studies, and integrating indicators of soil physicochemical properties, a database and evaluation system for soil-curing agent compatibility can be established. This will facilitate the rapid and accurate selection of the most suitable curing agent types and formulations for specific engineering needs and application scenarios, thereby optimizing the use of magnesium-based cementitious materials in soil curing projects. This approach aims to minimize costs while ensuring effective curing, and to enhance the economic and environmental benefits of such projects.

Therefore, despite the numerous advantages of magnesium-based cementitious materials in soil curing applications, their widespread and efficient utilization requires addressing current key issues such as unstable hydration products, unclear curing mechanisms, and limited soil applicability. By employing interdisciplinary research methodologies and fostering continuous technological innovation, the full potential of magnesium-based cementitious materials can be realized. This is expected to pave the way for advancements in soil curing technology, promoting its sustainable application in various fields such as infrastructure construction, geological disaster prevention and control, and land reclamation. Ultimately, this will provide robust technical support for socio-economic development.

## 10. Conclusions

This paper reviews several studies on magnesium-based cementitious materials, with a focus on their hydration products, performance characteristics, and application in soil stabilization. MOC stabilizes soil by forming 5·1·8/3·1·8 phase hydration product networks in pores to boost strength/durability, while ionic interactions adjust soil particle surface charges for better adhesion; admixtures like ammonium dihydrogen phosphate enhance compressive strength, and fly ash/sulfates improve water resistance. MOS curing relies on the 5·1·7 phase for strength, with Al_2_O_3_/Fe_2_O_3_/SiO_2_ admixtures creating the M-F-A-S gel phase filling the crystal lattice to enhance water resistance. MPC research focuses on immobilizing heavy metal elements Pb/Cd in contaminated soil. MPC-cured soil forms a unique three-dimensional structure, which enhances the reconstruction of the soil skeleton and the cementation of particles, thereby increasing the unconfined compressive strength. The environmental impact of MBCMs during the soil curing process is predominantly positive, exhibiting a significant reduction in CO_2_ emissions compared to traditional Portland cement-based binder. Future research should prioritize investigating the long-term durability of MBCMs in complex soil environments and developing unified application guidelines for different soil types, while practical implementation should emphasize scaling up field trials with industrial waste incorporation to optimize cost-effectiveness and environmental benefits.

## Figures and Tables

**Table 1:** Summary of various soil stabilization materials.

Soil Stabilizer Type	Soil Stabilization Materials	Soil Type	Solidification Mechanism	Solidification Effect	References
Inorganic Soil Stabilizers	Cement	Fly ash soil	Hydration products (calcium silicate gel and calcium hydroxide) fill the voids between soil particles and bond them together, thereby enhancing soil strength and stability	When the cement content was 12%, the 28-day UCS of the solidified soil reached 6.32 MPa, representing a 96.9% increase	[26]
	Portland cement and B-nZVI (bentonite-supported nano zero-valent iron) composites	Pb-contaminated soil	Physical encapsulation and chemical adsorption effects of the hydration products generated by the pozzolanic and hydration reactions of cement	When the cement content was 12% and the B-nZVI dosage was 1%; the leaching amount of Pb^2+^ decreased from 67.6 mg/kg to 6.59 mg/kg after 7 days	[27]
	Portland cement, granulated blast furnace slag, and sodium sulfate composites	Seaside silt	Formation of a significant amount of ettringite in the soil tightly bond soil particles and enhance soil strength	28-day UCS exceeded 5 MPa; reduced Cd^2+^ solubility	[28]
	Lime	Fine-grained soil	${Ca}^{2+}+{OH}^{-}+Soluble\;Clay\;Silica\to Calcium\;Silicate\;Hydrate\;(CSH)$ ${Ca}^{2+}+{OH}^{-}+Soluble\;Clay\;Alumina\to Calcium\;Alumina\;Hydrate\;(CAH)$	Ratio of soil strength in water-immersion-to-non-immersion conditions reaches ~0.8. Pre-compression value increases from 36 kN/m^2^ to 82 kN/m^2^; compression index decreases from 0.85 to 0.36	[29]
	3% raw lime, 3% limestone powder, and 3% WSS (slag-nanosilica stabilizer)	Drill cutting waste soil	Raw lime (CaO) hydrates with water to form Ca(OH)_2_, which reacts with soil’s acidic components (e.g., clay aluminosilicates) to generate cementitious compounds	28-day UCS reached 2.13 MPa, liquid limit decreased to 49.1%, plasticity index reduced to 14.8	[30]
	Water glass	Sand	A silica gel layer forms on soil particles, which bonds them effectively due to its superior adhesive properties	As the consolidation period extended from 3 days to 14 days, the strength of the sand soil increased from 300~1000 KPa to 4000~5600 KPa	[31]
	Water glass	Sulfate saline soil		Wave velocity of soil treated with 20°Bé water glass increased by 27.3% while that treated with 40° Bé water glass increased by 50.6%	[32]
	Modified water glass	Saline soil		At 20 °C, particles smaller than 10 μm exhibited significant agglomeration, thereby enhancing inter-particle adhesion and consequently increasing the compressive strength of the soil	[33]
Organic Soil Stabilizers	Asphalt	Silt	When mixed with soil, it coats soil particles, forming a continuous and flexible bonding system	Optimal water content for maximum performance was 14.0%, with an expansion rate controlled at 5.0%; California bearing ratio value was 159.15%	[34]
	Asphalt	Sand		Elastic modulus increased 3–7 times under single- and double-impact shear stresses. After 1200 and 1800 cycles of loading, the elastic modulus decreased by 32~61% and 34~63%, respectively	[35]
	Epoxy resin	Silty clay	Forming a robust polymer network between soil particles, firmly fixing them within the network	When the ratio of epoxy resin to water was 3:2, the stabilized soil exhibited a high shear strength of 226.4 kPa, with cohesion increasing to 182.1 kPa.	[36]
	Epoxy resin	Copper-contaminated soil	Porosity of the contaminated soil decreased, with epoxy resin filling the soil pores, thereby altering the soil structure and effectively inhibiting the diffusion of copper ions from the pores	14-day UCS of the contaminated soil reached 350 kPa	[37]
Biological Soil Stabilizers	MICP + fiber mixtures	Sandy soil	Utilizes specific microorganisms, such as Bacillus pasteurii, which induce the precipitation of calcium carbonate through their metabolic activities by utilizing calcium and carbon sources from the surrounding environment. These calcium carbonate precipitates accumulate between soil particles, acting as a binder to cement them together	After multiple MICP treatments, the surface structure of the samples became denser, allowing more calcium carbonate to fill pores and bond soil particles	[38]
	MICP + nano-silica	Clay mineral surfaces		Under 30% moisture content, the UCS of samples treated with MICP combined with 1.5% nano-silica reached 120 kPa, which was 6 times higher than untreated samples and 15 times higher than samples treated only with MICP	[39]
	MICP (microbial-induced calcite precipitation)	Sandy soil		At 7 days, the waterproofing rates for 0.315 mm, 0.63 mm, and mixed particle size MICP-treated samples were 2.8, 2.6, and 2.4, respectively; the scouring rates were 70, 50, and 60, respectively	[40]

**Table 2:** Application of MOC-based curing materials in different soils.

MOC Based Curing Agent Composition	Soil Type	Properties	Ref.
MgO 2%, brine concentration 4.67%, brine Dosage 3.2%, Mg/Cl = 35	Gravel soil	UCS 3.34 MPa	[57]
Light-burnt magnesium powder dosages 3%, brine dosing 8%	Gravel soil	Compacted material dry density 2.344 g/cm^3^	[58]
Oily sludge/MOC = 1.0 MgO/MgCl_2_: 2.0~3.0	Sludge	UCS exceed 11.20 MPa	[59]
MOC and lime, MgO/MgCl_2_ molar ratio 10	Sludge	UCS 1.9 MPa	[60]
MgO: MgCl_2_: H_2_O = 2.45: 1: 14 to 6.3: 1: 14	Subgrade soil	UCS remains 1.26 MPa immersed in water for 24 h	[61]
MgO/MgCl_2_ molar ratio 8.61, MOC content 18%, fly ash content 20.36%	Pavement base and sub-base soil	UCS 2.56 MPa, softening coefficient 0.76	[62]
MOC, 1% ammonium dihydrogen phosphate	Waste soil	5.31 MPa, softening coefficients 0.98–0.95	[63]

**Table 3:** Application of MOS-based curing materials in different soils.

MOS-Based Curing Agent Composition	Soil Type	Properties	Ref.
TZ18 (MOS–water glass–clinker–silica fume = 3.5: 1.2: 1.0: 1.1)	Muddy soft soil	7d UCS 879.7 kPa	[5]
Dosage of humic acid *W*_h_ (6%), the initial soil–water content *W*_w_ (60%); MMOS dosage *W*_m_ (18%); optimal mixing dosages: sodium silicate, silica fume, and cement clinker, 2–5%, 2–6%, and 2–6%	Marine organic silt	Maximum error of predicted *UCS* is merely 4.38%	[75]
Citric acid-modified magnesium oxysulfate cement, silica fume, and clinker	Marine soft clay	5·1·7 phase, gel, dolomite, pyrophyllite, a few CaO and MgO; 7d UCS 435.81 kPa (stabilizing agent 5%); 842.64 kPa (stabilizing agent 20%)	[76]
Magnesium oxysulfate cement composite curing agent	Mudflat soft clay with high soil moisture and low compression modulus	The smaller the initial soil moisture, the larger the amount of curing agent and the longer the age, the greater initial tangent modulus and shear strength parameters of the solidified soil	[72]
10% alkaline magnesium sulfate cement, 1% citric acid, 4% water glass, 12% fixed sulfur ash, 8% silica fume	Muddy soft soil	28d UCS 1400 kPa	[77]
11% magnesium oxysulfate cement	Loess in Yan’an	28d UCS 9.4 MPa, softening coefficient 0.947 soaking in water 24 h	[74]

**Table 4:** Application of MPC-based curing materials in different soils.

MPC Based Curing Agent Composition	Soil Type	Properties	Ref.
MPC addition from 30 to 70%; water-soil ratio 0.45	lead contaminated soil	UCS increased from 0.15 MPa to 0.67 MPa; destructive strain (ε_f_) decreased from 8.4 to 1.8%; lead content 500 mg/kg	[96]
hydrogen peroxide-based MPC	fluoride-contaminated soil	Optimal adsorption capacity for fluoride reaching 2.21 mg/g; pore volume from 0.112 cm^3^/g to 0.080 cm^3^/g; remained stable and intact after 15 days	[97]
MPC with varying types of activated magnesium oxides; optimal ratio dead burnt magnesia: light burnt magnesia = 3:7	waste sludge	28d UCS approximately two to three times greater than that at 14 days; strength retention rate remained 79~96% after being submerged in water for 28d days	[98]
MKPC mixed with 30% silica fume	municipal sludge	7d UCS reached 430 kPa, exceeds the minimum requirement for landfill materials (≥350 kPa); moisture content reduced to less than 30%; volumetric shrinkage within 7 days	[99]
hydration cementation products of phosphate as the binding phase, MgO crystals as the skeletal framework	water purification sludge	28d UCS of the sludge cured with a 40% MPC dosage reached 1584 kPa	[100]

## Data Availability

The authors confirm that the data supporting the findings of this study are available within the article.

## References

[1] Hu J., Wang Y., Zhang S., Chang T., Sun L. (2023). Preparation, classification, hydration mechanism and durability of magnesium-based cementing material. J. Min. Sci. Technol..

[2] Di S. (2001). China’s magnesite resources and market. Non-Met. Miner..

[3] Dong J., Wen J., Zheng W., Jia L., Chang C., Xiao X. (2023). Heat treatment technology of salt lake magnesium slag and its influence on the properties of magnesium phosphate cement. Mater. Rep..

[4] Zeng T., Chen X., Wang N., Zhang T., Guan Y., Bi W., Chang J. (2024). Review of magnesium cementitious material: Current developments and low carbon path. Mater. Rep..

[5] Zhu J., Rao C., Tuo Q., Liu H., Pan B. (2019). Experimental study on the properties of the organic soil solidified by the composite magnesium oxysulfate cement-curing agent. Chin. J. Rock Mech. Eng..

[6] Hang Z. (2024). The current situation and thoughts on the development of green construction in china’s building engineering. Constr. Saf..

[7] Li S., Du S., Bao S., Yan S., Liu Z. (2023). Bibliometric analysis and development trend discussion of contaminated soil remediation technology in international research. Environ. Eng..

[8] Liu J., Ye Z., Guan B., Fang J. (2018). Research progress on rapid repair materials of magnesium phosphate cement. Hans J. Civ. Eng..

[9] Chen W., Xiao X., Zhang L., Dong J., Zheng W. (2022). Research on low carbon development and application of magnesia cement--low carbon products and technology cases of green building materials. Constr. Technol..

[10] Loye H.-C.Z., Vecernik P., Kiselova M., Kašpar V., Korenkova H., Miller V., Bezdicka P., Šubrt J., Murafa N., Shkuropatenko V. (2024). Investigation of Magnesium-Potassium Phosphates as Potential Nuclear Waste Form for the Immobilization of Minor Actinides. Inorganics.

[11] Yuan Y., Zhang Q., Lin S., Li J. (2024). Water: The soul of hydrogels. Prog. Mater. Sci..

[12] Runčevski T., Wu C., Yu H., Yang B., Dinnebier R.E., Jennings H. (2013). Structural Characterization of a New Magnesium Oxysulfate Hydrate Cement Phase and Its Surface Reactions with Atmospheric Carbon Dioxide. J. Am. Ceram. Soc..

[13] Dinnebier R.E., Pannach M., Freyer D. (2013). 3Mg(OH)_2_•MgSO_4_•8H_2_O: A metastable phase in the system Mg(OH)_2_-MgSO_4_-H_2_O. Z. Anorg. Allg. Chem..

[14] Gardner L.J., Walling S.A., Lawson S.M., Sun S., Bernal S.A., Corkhill C.L., Provis J.L., Apperley D.C., Iuga D., Hanna J.V. (2020). Characterization of and Structural Insight into Struvite-K, MgKPO_4_·6H_2_O, an Analogue of Struvite. Inorg. Chem..

[15] Yuan G., Zhang J., Liu G. (2023). Experimental Investigation and Field Evaluation of Anti-Scouring and Protection Material of Earth-rock Dam. KSCE J. Civ. Eng..

[16] Cheng Y., Yu H., Zhu B.-L., Wei D.-X. (2016). Laboratory investigation of the strength development of alkali-activated slag-stabilized chloride saline soil. J. Zhejiang Univ. A.

[17] Cao C., Li G. (2023). Stabilizing soil using annealed polyvinyl alcohol as long-lasting binder. arXiv.

[18] Consoli N.C., Caicedo A.M.L., Saldanha R.B., Filho H.C.S., Acosta C.J.M. (2020). Eggshell Produced Limes: Innovative Materials for Soil Stabilization. J. Mater. Civ. Eng..

[19] Chung H., Kim S.H., Nam K. (2020). Application of microbially induced calcite precipitation to prevent soil loss by rainfall: Effect of particle size and organic matter content. J. Soils Sediments.

[20] Khan S., Naushad M., Lima E., Zhang S., Shaheen S., Rinklebe J. (2021). Global soil pollution by toxic elements: Current status and future perspectives on the risk assessment and remediation strategies–A review. J. Hazard. Mater..

[21] Sörengård M., Kleja D.B., Ahrens L. (2019). Stabilization and solidification remediation of soil contaminated with poly- and perfluoroalkyl substances (PFASs). J. Hazard. Mater..

[22] Taharia M., Dey D., Das K., Sukul U., Chen J.-S., Banerjee P., Dey G., Sharma R.K., Lin P.-Y., Chen C.-Y.J.E. (2024). Microbial induced carbonate precipitation for remediation of heavy metals, ions and radioactive elements: A comprehensive exploration of prospective applications in water and soil treatment. Ecotoxicol. Environ. Saf..

[23] Ghodousian O., Ghodousian A., Shafaie V., Hajiloo S., Movahedi Rad M.J.B. (2023). Study of bonding between façade stones and substrates with and without anchorage using shear-splitting test—case study: Travertine, Granite, and Marble. Build..

[24] Miller Sabbie A., Myers Rupert J. (2020). Environmental Impacts of Alternative Cement Binders. Environ. Sci. Technol..

[25] Madlool N., Saidur R., Hossain M., Rahim N. (2011). A critical review on energy use and savings in the cement industries. Renew. Sustain. Energy Rev..

[26] Li C., Wang X., Gao G. (2024). Engineering performance analysis of stabilized fly ash-soil with curing agent. J. Wuhan University Technol..

[27] Yu C., Yu Z.-K., Liao R.-P., Wang Y.-B., Cai X., Zeng Z.-L. (2024). B-nZVI optimization of strength and heavy metal stability of lead-contaminated soil solidified by Portland cement. Environ. Geochem. Health.

[28] Zhang G., Wang B., Zhang H., Zhang S., Kang M. (2022). Effects of OPC-GBFS-NS system based soil stabilizer on soil stabilization. J. Build. Mater..

[29] Jawad I.T., Taha M.R., Majeed Z.H., Khan T.A. (2014). Soil stabilization using lime: Advantages, disadvantages and proposing a potential alternative. Res. J. Appl. Sci., Eng. Technol..

[30] Wu Y., Wang X., Jin S., Dong B., Liu Y. (2023). Application of quicklime-limestone powder-WSS soil stabilizer in road regeneration of drilling waste mud. J. Eng. Geol..

[31] Xu Y. (2020). Experimental Study on Sodium Silicate Reinforced Sand. Master’s Thesis.

[32] Chen H. (2018). Study on Triaxial Tests of Water Glass Solidified Sulphuric Acid Saline Soil. Master’s Thesis.

[33] Lv Q., Meng H., Wang S., Zhang Q., Chen H. (2017). Research of strength and freezing-thawing durability of saline soil solidified by modified sodium silicate. J. Beijing Univ. Technol..

[34] Zhang Q., Zhang L., Wang X., Sun Z., Zhao Q. (2021). Comparative experimental study on cbr performance of different chemically modified soil. Mater. Sci..

[35] Sarsam S.I., Kais A.T. (2019). Resilient characteristics of asphalt stabilized soil. Sustain. Civ. Infrastruct..

[36] Liu Z. (2021). Mechanical Properties of Silty Clay Reinforced by Epoxy Resin and Bamboo Slices. Master’s Thesis.

[37] Ma Q., Lei J., He J., Chen Z., Li W. (2023). Epoxy resin for solidification/stabilization of soil contaminated with copper (II): Leaching, mechanical, and microstructural characterization. Environ. Res..

[38] Xiao Z. (2021). Experimental Study on Soil Consolidation by Improved Microbial-Induced Calcium Carbonate Precipitation. Master’s Thesis.

[39] Ghalandarzadeh S., Maghoul P., Ghalandarzadeh A., Courcelles B. (2024). Effect of nanoparticle-enhanced biocementation in kaolinite clay by microbially induced calcium carbonate precipitation. Constr. Build. Mater..

[40] Huynh N., Huyen N., Truong H., Son N. (2023). A study on the applicability of microbially induced calcium carbonate precipitation on soil-sand stabilization through the bio-cementation process. IOP. Conf. Ser. Earth Environ. Sci..

[41] Wang L., Li D. (2018). Preparation of high strength and high toughness magnesium oxychloride cement based cementitious material. Bull. Chin. Ceram. Soc..

[42] Urwongse L., Sorrell C.A. (1980). The system MgO-MgCl_2_-H_2_O at 23-degrees-c. J. Am. Ceram. Soc..

[43] Sglavo V.M., De Genua F., Conci A., Ceccato R., Cavallini R. (2011). Influence of curing temperature on the evolution of magnesium oxychloride cement. J. Mater. Sci..

[44] Wu J., Chen H., Guan B., Xia Y., Sheng Y., Fang J. (2019). Effect of Fly Ash on Rheological Properties of Magnesium Oxychloride Cement. J. Mater. Civ. Engin..

[45] Yu G. (1993). Discussion on several issues concerning magnesium oxide chloride cement products. Constr. Saf..

[46] Chen X., Bi W., Zhang T., Yu H., Guan Y., Liang Y. (2019). Modification of magnesium oxychloride cement by citric acid. J. Chin. Ceram. Soc..

[47] Liang X. (2023). Preparation and Performance Evaluation on Fireproof Lightweight Magnesium Oxychloride Cement-Based Waste Wood Composite Panel. Master’s Thesis.

[48] Feng K., Wang L., Chen X. (2015). Research on improving the water resistance of magnesium oxychloride cement. J. Funct. Mater..

[49] Deng D. (2003). The mechanism for soluble phosphates to improve the water resistance of magnesium oxychloride cement. Cem. Concr. Res..

[50] Wang L. (2012). Research on the compound modification on magnesium oxychloride cement. J. Funct. Mater..

[51] Cao H., Li Y., Feng Q., Xue C., Wang P., Qiao H. (2024). Analysis on long-term durability and degradation law of coated reinforced magnesium oxychloride cement concrete. J. Mater. Sci. Eng..

[52] Li J. (2023). Research on the Seawater Corrosion Resistance of Magnesium Oxychloride Cement. Master’s Thesis.

[53] Zheng W., Chang C., Xiao X., Zhou Y., Dong J., Li Y., Wen J., Huang Q., Man Y., A D. (2019). Study on applicability of foaming agent to magnesium oxychloride cement. J. Salt Lake Res..

[54] Ma J., Zhao Y., Wang J., Wang L. (2010). Effect of Magnesium Oxychloride Cement on Stabilization/Solidification of Sewage Sludge. Constr. Build. Mater..

[55] Wang D., Di S., Gao X., Wang R., Chen Z. (2020). Strength properties and associated mechanisms of magnesium oxychloride cement-solidified urban river sludge. Constr. Build. Mater..

[56] Wang D., Gao X., Liu X., Zeng G. (2021). Strength, durability and microstructure of granulated blast furnace slag-modified magnesium oxychloride cement solidified waste sludge. J. Clean. Prod..

[57] Li Y., Xiao X., Wen J., Huang Q., Chang C., Dong J., An S., Zheng W. (2017). Influence of raw material ratio of magnesium oxychloride cement on the compressive strength of solidified gravel soil. Chin. J. Rock Mech. Eng..

[58] Xiao X., Chang C., Li Y., An S., Wen J., Zheng W., Huang Q., Dong J. (2018). The Influence of raw material ratio of magnesium oxychloride cement on dry density of solidified gravel soil. J. Salt Lake Res..

[59] Li H., He H., Liang J., Wang L., Zhang Y. (2024). Solidification/stabilization of oily sludge using magnesium oxychloride cement. Liaoning Chem. Ind..

[60] Wang Q., Cheng Y., Cai G., Guo J., Li Y. (2024). Experimental analysis of mechanical properties of dredged sludge solidified by carbonized under the synergistic action of magnesium oxychloride cement and lime. Constr. Build. Mater..

[61] Wang H., Zhang J., Yan X., Xiong R., Rangasamy B. (2022). Study on Properties of Magnesium Oxychloride Cement Solidified Soil. Adv. Mater. Sci. Eng..

[62] Zhang H., Wang K., Hu B., Zheng Y., Tang S. (2023). Study on Proportion Optimization of Magnesium Oxychloride Cement-Stabilized Clayey Soil Based on the Response Surface Methodology. Adv. Mater. Sci. Eng..

[63] Li C., Wang W., Peng J., Liang Z., Dou R., Zhang S. (2024). Research on modification and properties of magnesium oxychloride cement-based fluid solidified soil. China Concr..

[64] Walling S.A., Provis J.L. (2016). Magnesia-based cements: A journey of 150 years, and cements for the future?. Chem. Rev..

[65] Demediuk T., Cole W., Hueber H. (1955). Studies on magnesium and calcium oxychlorides. Aust. J. Chem..

[66] Urwongse L., Sorrell C.A. (1980). Phase Relations in Magnesium Oxysulfate Cements. J. Am. Ceram. Soc..

[67] Yu H., Wu C., Wang C. The theoretical innovation of basic magnesium sulfate cement and its application prospect of foamed concrete. Proceedings of the 2013/2014 CCPA China foam Concrete Annual Conference and National Foam Concrete Technology Exchange Conference.

[68] Zhu X., Zhu Q., Zhu Y., Zhu Y. (2022). Experimental study on the effect of hydrophobizing agent on the properties of magnesium oxysulfate cement concrete. Shanghai Build. Mater..

[69] Wu C., Xing S., Zhang W., Jiang N., Zhang H., Yu H. (2016). Study on hydration mechanism of basic magnesium sulfate cement. J. Funct. Mater..

[70] Wang R., Ji X., Zhou R., Jin C., Sun W., Wang Z., Yan Y. (2024). Fabrication of high-strength, water-resistant homogeneous magnesium oxysulfate cement via synergistic modification with citric acid and sodium alginate. Constr. Build. Mater..

[71] Ba M., Zhu J., Xue T., Liu J. (2018). Influence of Molar ratio on properties of magnesium oxysulfate cementitious materials. J. Build. Mater..

[72] Zhu J., Yang H., Xu R., Pan B., Rao C. (2022). Mechanism of mudflat soft clay stabilized by magnsia oxysulfate cement and analysis on its micro-behavior for offshore wind farm. Acta Energ. Sol. Sin..

[73] Yan X., Xu Q., Deng M., Sun Y., He X., Dong S., Ma L., Hai C., Zhou Y. (2024). Investigation on the solidification effect and mechanism of loess utilizing magnesium oxysulfate cement as a curing agent. Sci. Total. Environ..

[74] Yan X., Xu Q., Dong S., Sun Y., Ma L., He X., Hai C., Zhou Y. (2024). Improved stabilization of loess soil using magnesium oxysulfate cement-based ecological composite binder. Constr. Build. Mater..

[75] Zhu J.-F., Tao Y.-L., Xu R.-Q., Yang H., Pan B.-J. (2022). Investigation on the optimal formulation and mechanism of marine organic silt improved with magnesium-cement-based stabilizer. Constr. Build. Mater..

[76] Zhu J.-F., Xu R.-Q., Zhao H.-Y., Luo Z.-Y., Pan B.-J., Rao C.-Y. (2020). Fundamental mechanical behavior of CMMOSC-S-C composite stabilized marine soft clay. Appl. Clay Sci..

[77] Wu J., Xu X., Xiao S., Cao K., Li X. (2024). Experimental study on the strength characteristics and microscopic mechanism of alkaline magnesium sulfate cement based solidification agent for solidifying silty soil. J. Wuhan Univ. Technol..

[78] Frantzis P., Baggott R. (2000). Bond between reinforcing steel fibres and magnesium phosphate/calcium aluminate binders. Cem. Concr. Compos..

[79] Wang H., Cao J., Xue M., Yang T. (2009). Study on the new phosphate cement repair material with super rapid hardening. New Build. Mater..

[80] Abdelrazig B., Sharp J. (1988). Phase changes on heating ammonium magnesium phosphate hydrates. Thermochim. Acta.

[81] Sugama T., Kukacka L.E. (1983). Characteristics of magnesium polyphosphate cements derived from ammonium polyphosphate solutions. Cem. Concr. Res..

[82] Xu C., Han J., Yang Y. (2024). A review on magnesium potassium phosphate cement: Characterization methods. J. Build. Eng..

[83] Xu Y., Deng L., Yang J., Zuo L., Du G., Lu Y., Li S. (2019). Preparation and rapid repair application of magnesium phosphate cement. Mater. Reports..

[84] Liu F., Pan B., Zhou C., Wang B., Nie J. (2024). Investigation of fire resistance of fly ash blended magnesium phosphate cement. China J. Highw. Transp..

[85] Yang Y., Liu Y., Yan Z., Chen Z. (2022). High-temperature resistance of modified potassium magnesium phosphate cement. Mater..

[86] Li X., Guo Y., Zhang Z., Meng X., Ji X. (2024). Investigation of factors influencing the compressive strength of magnesium phosphate cement mortars. Ind. Constr..

[87] Chen X., Guo J., Yu H., Zhang G., Kang Y., Zhang M., Hao T., Du Q. (2024). Impact of calcination temperatures on lithium magnesium slag for enhanced magnesium phosphate cement properties. Constr. Build. Mater..

[88] Shu Q. (2024). Study on mechanics and durability of sintered mud ash modified magnesium phosphate cement. Shanxi Archit..

[89] Sun J., Wang H., Lan J., Li Y., Cao Z. (2019). Analysis of the effect of additives on water resistance of magnesium phosphate cement. J. Hebei Instit. Archit. Civ. Eng..

[90] Chen X., Liu W., Cui A., Zheng H., Huang X., Yang W., Ge Y. (2024). Mechanical properties and freeze-thaw cycling resistance of magnesium phosphate cement mortar prepared at low temperatures in highland regions. Mater. Rep..

[91] Yuan J., Huang X., Chen X., Ge Q., Zhang Z. (2022). Early-age mechanical properties and hydration degrees of magnesium phosphate cement paste in freezing winter of cold regions. Constr. Build. Mater..

[92] Wang Z., Ding Y., Xu S., Xiong Z., Zhou H., Wu X. (2017). Semi-dynamic leaching tests on leaching properties of MPC-solidified zinc-contaminated soil under acid rain environment. Chin. J. Geotech. Eng..

[93] Wang P., Xue Q., Li J., Zhang T., Wang S., Li Z., Liu L. (2018). Factors affecting the leaching behaviours of magnesium phosphate cement-stabilised/solidified Pb-contaminated soil, part 1: Water-to-solid ratio and Pb concentration. Int. J. Environ. Pollut..

[94] Fan X., Wang B., Tang S., Xin X., Pei Y. (2024). Study on pH, conductivity response and pore characteristics of low liquid limit silt reinforced by magnesium phosphate cement. Mater. Rep..

[95] Feng Y.-S., Du Y.-J., Reddy K.R., Xia W.-Y. (2018). Performance of two novel binders to stabilize field soil with zinc and chloride: Mechanical properties, leachability and mechanisms assessment. Constr. Build. Mater..

[96] Zhang T., Li J., Wang P., Li Z. (2016). Experimental study of stress-strain properties of lead-contaminated soils treated by magnesium phosphate cement. Rock Soil Mech..

[97] Sana G., Abdulrahman A., Bechir H., Mohamed K., Clarence C., Mahmoud C. (2023). Synthesis and characterization of new composite materials based on magnesium phosphate cement for fluoride retention. Materials.

[98] Peng L., Chen B. (2021). Study on the basic properties and mechanism of waste sludge solidified by magnesium phosphate cement containing different active magnesium oxide. Constr. Build. Mater..

[99] Chen Y., Wang L., Song P., Wang Q. (2017). Effects of magnesium potassium phosphate cements mixed with silica fume on the solidification and reduction of municipal sludge. IOP Conf. Ser. Mater. Sci. Eng..

[100] Zhong L., Dai J., Lei Z., Feng D. (2024). Experimental study on water purification sludge solidified with magnesium phosphate cement. Guangzhou Archit..

[101] Cai G. (2017). Experimental and Application Studies on Soft Soil Carbonated and Stabilized by Reactive Magnesia. Ph.D. Thesis.

[102] Li W., Sun Z., Zhuang Y., Xiao H., Fu Z., Zhou X. (2024). Mechanical and swelling properties, as well as micro-mechanism of sulfate-bearing soil stabilized by magnesium oxide and cement. Chin. J. Geotech. Eng..

[103] Yuan X., Xiong T., Wang H., Wu Z., Jiang L., Zeng G., Li Y. (2018). Immobilization of heavy metals in two contaminated soils using a modified magnesium silicate stabilizer. Environ. Sci. Pollut. Res..

[104] Xu P., Zhu J., Gao X., Kang Z., Yang J. (2013). Study on the process of the preparation of activated MgO by calcining magnesite. Multipurp. Util. Miner. Resour..

[105] Tan Y., Liu S., Achintha M., Mi R. (2024). Is It Possible to Make Magnesia-Based Cement Environmentally Friendly?. ACS Sustain. Chem. Eng..

[106] Tan Y., Yu H., Li Y., Dong J., Wen J., Wu C., Xiao W. (2014). Preparation of fly ash magnesium potassium phosphate cement using byproduct magnesium oxide containing boron from salt lakes. J. Chin. Ceram. Soc..

[107] Tan Y., Yu H., Li Y., Bi W., Yao X. (2016). The effect of slag on the properties of magnesium potassium phosphate cement. Constr. Build. Mater..

[108] Liska M., Al-Tabbaa A. (2009). Ultra-green construction: Reactive magnesia masonry products. Proc. Inst. Civ. Eng.–Waste Resour. Manag..

[109] Ma C., Chen G., Shi J., Zhou H., Ren W., Du Y. (2022). Improvement mechanism of water resistance and volume stability of magnesium oxychloride cement: A comparison study on the influences of various gypsum. Sci. Total. Environ..

[110] Wang J., Ma K., Shang S., Ran J., Xia H., Luo Z., Li Y., Shi Y., Chen G., Tang Z. (2024). Exploration of the effects of electrolytic manganese residue on the environmental, economic, and engineering performance of magnesium oxychloride cement: A possible utilization method of electrolytic manganese residue. Constr. Build. Mater..

[111] Ping C. (2016). Study on New Technology of Preparation of Potassium Dihydrogen Phosphate with Wet Process Phosphoric Acid. Master’s Thesis.

